# Longitudinal Exposomics in a Multiomic Wellness Cohort Reveals Distinctive and Dynamic Environmental Chemical Mixtures in Blood

**DOI:** 10.1021/acs.est.4c05235

**Published:** 2024-09-05

**Authors:** Kalliroi Sdougkou, Stefano Papazian, Bénilde Bonnefille, Hongyu Xie, Fredrik Edfors, Linn Fagerberg, Mathias Uhlén, Göran Bergström, Leah J. Martin, Jonathan W. Martin

**Affiliations:** † Department of Environmental Science, Stockholm University, Stockholm 106 91, Sweden; ‡ National Facility for Exposomics, Metabolomics Platform, Science for Life Laboratory, Stockholm University, Solna 171 65, Sweden; § Department of Protein Science, Science for Life Laboratory, KTH-Royal Institute of Technology, Stockholm 100 44, Sweden; ∥ Department of Molecular and Clinical Medicine, Institute of Medicine, Sahlgrenska Academy, University of Gothenburg, Gothenburg 40530, Sweden; ⊥ Department of Clinical Physiology, Sahlgrenska University Hospital, Region Västra Götaland, Gothenburg 413 45, Sweden; # Independent Researcher, Stockholm 112 33, Sweden

**Keywords:** chemical exposome, longitudinal exposomics, high-resolution mass spectrometry, multiclass targeted, untargeted analysis, blood plasma

## Abstract

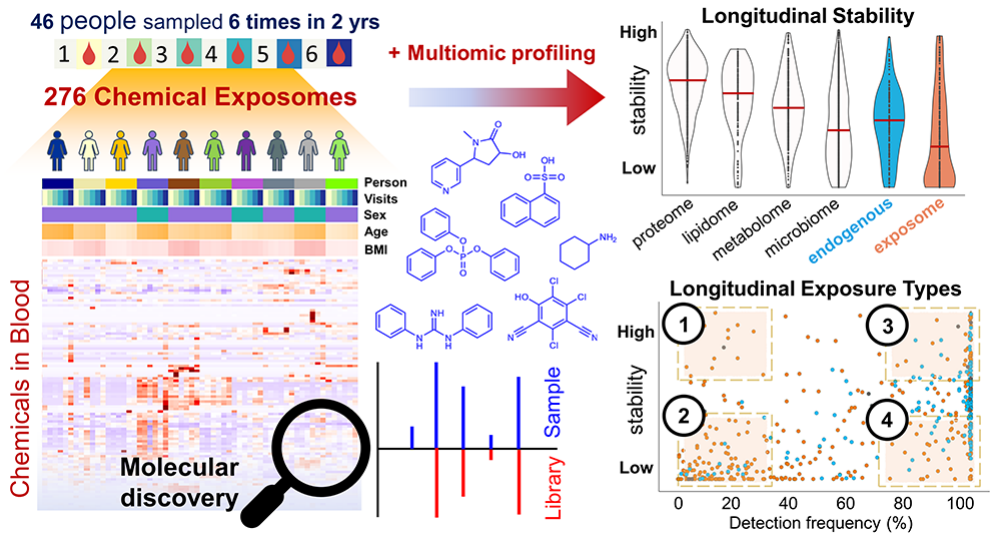

Chemical exposomes can now be comprehensively measured in human blood, but knowledge of their variability and longitudinal stability is required for robust application in cohort studies. Here, we applied high-resolution chemical exposomics to plasma of 46 adults, each sampled 6 times over 2 years in a multiomic cohort, resulting in 276 individual exposomes. In addition to quantitative analysis of 83 priority target analytes, we discovered and semiquantified substances that have rarely or never been reported in humans, including personal care products, pesticide transformation products, and polymer additives. Hierarchical cluster analysis for 519 confidently annotated substances revealed unique and distinctive coexposures, including clustered pesticides, poly(ethylene glycols), chlorinated phenols, or natural substances from tea and coffee; interactive heatmaps were publicly deposited to support open exploration of the complex (meta)data. Intraclass correlation coefficients (ICC) for all annotated substances demonstrated the relatively low stability of the exposome compared to that of proteome, microbiome, and endogenous small molecules. Implications are that the chemical exposome must be measured more frequently than other omics in longitudinal studies and four longitudinal exposure types are defined that can be considered in study design. In this small cohort, mixed-effect models nevertheless revealed significant associations between testosterone and perfluoroalkyl substances, demonstrating great potential for longitudinal exposomics in precision health research.

## Abstract 2

The first cohort-level application of longitudinal exposomics revealed novel and dynamic coexposures in blood of relevance to precision health.

## Introduction

The chemical exposome represents the cumulative sum of environmental chemical exposures throughout an individual’s life course. It includes exposure to natural and anthropogenic chemicals from external sources, such as inhalation of polluted air, intake of dietary substances and pharmaceuticals, and ingestion of contaminated food and water, but also includes internal exposure sources, such as the metabolic products of gut microbiota.^1,2^ With recognition that our environment is dynamic over time and that our susceptibility to disease changes over the life course, the exposome has always been imagined as a longitudinal endeavor that will require multiple measures of exposure.^1,2^ For example, the external airborne chemical exposome has been shown to be highly dynamic for individuals over time, and variable among people, even for those living in the same geographical area.^3^

Comparatively little is known about the dynamics and variability of chemical exposomes in human blood. Sensitive methods for chemical exposomics have been described for blood plasma, including by multiclass targeted analysis^4,5^ and combined targeted/untargeted analysis by liquid chromatography-high-resolution mass spectrometry (LC-HRMS).^6,7^ Measurement of the chemical exposome in blood is strategically important because the same sample can be further used for clinical testing, or for multiomic profiling of endogenous molecules whose levels may be impacted by the exposome.^8,9^ Previous longitudinal studies of small molecules in blood have focused on the metabolome, with limited exploration of environmental chemicals in a few individuals for up to a few months.^9,10^ Studies of the blood chemical exposome have yet to be reported in a longitudinal cohort, and consequently, there are few measures of inter- and intraindividual variability for priority environmental substances. Such data are necessary to differentiate short-term and chronic exposures and to inform the number of blood sampling events to include in statistically powered exposome studies in the future.

Here, we applied chemical exposomics to recurrent plasma samples of 46 healthy Swedish adults, each sampled 6 times over 2 years in a multiomic wellness profiling study. Using LC-HRMS for quantitative analysis of 83 multiclass targeted analytes (78 priority environmental contaminants and 5 steroid hormones) and parallel untargeted discovery of environmental chemicals and endogenous metabolites,^7^ here we report the inter- and intraindividual variability for hundreds of molecular environmental exposures, including chemicals not previously detected in human blood. Intraclass correlation coefficients (ICCs) revealed that the stability of the chemical exposome was generally low, compared to parallel measures of plasma proteome, metabolome, lipidome, and microbiome in the same participants. Repeated sampling of the same individuals over time permitted new exposure types to be defined, and for rare and common coexposures to be revealed of relevance to precision health. Mixed-effect modeling also revealed statistically significant exposome-metabolome interactions indicative of endocrine disruption.

## Methods

### Recruitment and Sampling

Plasma samples were from healthy participants in the Swedish SciLifeLab SCAPIS Wellness Profiling (S3WP) study^11^ who were previously recruited to the Swedish CArdioPulmonary bioImage Study (SCAPIS), a cohort of 30,000 participants aged 50–65 years and representative of the Swedish population.^11,12^ S3WP included 6 examination visits in two rounds (4 visits scheduled every 3 months in the first round, and 2 visits scheduled every 6 months in the second round), with 94 of 101 enrolled subjects completing all 6 visits between late 2015 and early 2018.^11^ At each visit and after overnight fasting, samples of blood, urine, and feces were collected.^11^ In this work, longitudinal sample sets of plasma from 46 participants (6 samples per person, 276 total samples, 50–200 μL) were selected so that 23 males and 23 females were included, with balanced birth years in the ranges 1950–1955 (*n* = 14; 7 males and 7 females), 1956–1960 (*n* = 16; 7 males and 9 females), and 1961–1965 (*n* = 16; 9 males and 7 females). The study was approved by the Ethical Review Board of Göteborg, Sweden, and all participants provided written informed consent.

### Sample Preparation and LC-HRMS Analysis

Plasma samples were prepared and analyzed following a previously described combined targeted and untargeted chemical exposomics method.^7^ The reader is directed to Sdougkou et al.^7^ for details on sample preparation, analysis, validation, and quantification; further information can be also found in the Supporting Information and Table S1. The chemical exposomics method was validated for 83 targeted analytes, including environmental contaminants, dietary chemicals, tobacco markers, drugs, and endogenous steroid hormones (Table S1).^7^ Sample preparation followed a phospholipid removal protocol, and measurements were conducted by ultrahigh pressure LC (Ultimate 3000, Thermo Scientific) with HRMS acquisition (Q Exactive Orbitrap HF-X, Thermo Scientific) in positive and negative electrospray ionization mode (ESI+ and ESI−).^7^ Spectral acquisition was performed with alternating full scan and data-independent MS/MS acquisition (DIA). Data-dependent acquisition (DDA) with an inclusion list of precursor ions was used for analyte confirmations. Quantification of target analytes was by solvent-based calibration curves with internal standards and by reference standardization,^13^ which was facilitated by injecting pooled Swedish plasma (see the Supporting Information), for retrospective semiquantification of discovered untargeted substances. For data summaries and statistics, when analytes were detected at concentrations lower than the respective MLOQ, concentrations were substituted by MLOQ/2, and when analytes were nondetect, concentrations were substituted by MLOQ/4.

### Untargeted Data Processing and Structural Annotations

For untargeted analysis and spectral library matching, raw data were processed in MS-DIAL (v.4.90)^14^ for feature alignment across samples, MS1 and DIA MS2 spectral deconvolution, and peak integration (Table S2). Each molecular feature was defined by a chromatographic retention time (RT), an MS1 *m*/*z*, and a deconvoluted MS2 spectrum. For feature annotation, spectral matches were considered for total identification scores >700 using MassBankEU,^15^ MassBank of North America,^16^ and Global Natural Product Social Molecular Networking (GNPS).^17^ For each annotated feature, a class (endogenous/environmental) and subclass (e.g., bile acids, PFAS) was assigned following searches of PubChem,^18^ Human Metabolome Database,^19^ and Food Metabolome Database.^20^

The MS-DIAL feature lists from ESI+ and ESI– were combined and analyzed in Python (v. 3.8.16)^32^ using Jupyter Notebook (v. 6.5.2).^33^ Redundant features across modes were identified based on mass tolerance of 0.002 Da (after adjusting for (de)protonation) and RT tolerance of 0.2 min, and the redundant feature with the lowest average peak area was discarded. Peak areas of the final combined data set were corrected for instrumental and batch variation using the principal component analysis (PCA) scores of isotope-labeled internal standard signal intensities in each sample,^21,22^ and then normalized by the analyzed sample volume (complete data reduction workflow in the Supporting Information).

### Statistical Analysis and Visualization

PCA was performed in SIMCA (v. 17.0, Umetrics) for the targeted data set. ComplexHeatmap (2.14.0)^23^ was used to visualize the combined targeted and annotated untargeted data set in R (v. 4.2.1) and R Studio (v. 2023.03.1). Hierachical cluster analysis (HCA) dendrograms and heatmaps were generated based on Pearson correlation as clustering distance and average linkage as the clustering similarity method. For other data visualizations, the Python libraries Plotly (v.5.13.0)^24^ and Seaborn (v.0.12.2)^25^ were used. The package SciPy (v. 1.7.3)^26^ was used for Shapiro-Wilk test, Wilcoxon signed-rank test, Student’s *t* test, one-way ANOVA, and Pearson correlation coefficient calculation. Statsmodels (v. 0.13.5)^27^ was used for Bonferroni corrections and Tukey’s test, Pingouin (v. 0.5.3)^28^ was used for the ICC calculations, and umap-learn (v. 0.5.3) was used for uniform manifold approximation and projection (UMAP).^29^ For UMAP, default parameters were used: number of neighbors (15), minimum distance (0.1), and Euclidean distance. Unit-variance scaling was applied before the UMAP or PCA analyses. Mixed-effect linear regression analyses were conducted with lme4 (v 1.1–33) in R (v 4.3.0) to examine associations between PFAS and testosterone; models included participant-specific random effects with random intercepts and slopes. Associations between log_10_ transformed testosterone and log_10_ transformed PFAS concentrations were examined in unadjusted models, as well as in models adjusted for baseline age and body mass index (BMI) as fixed effects, and in both cases the *p*-values were Bonferroni corrected for multiple hypotheses using ‘*p*.adjust’ in R.

## Results and Discussion

### Multiclass Targeted Analysis

**Figure 1:**
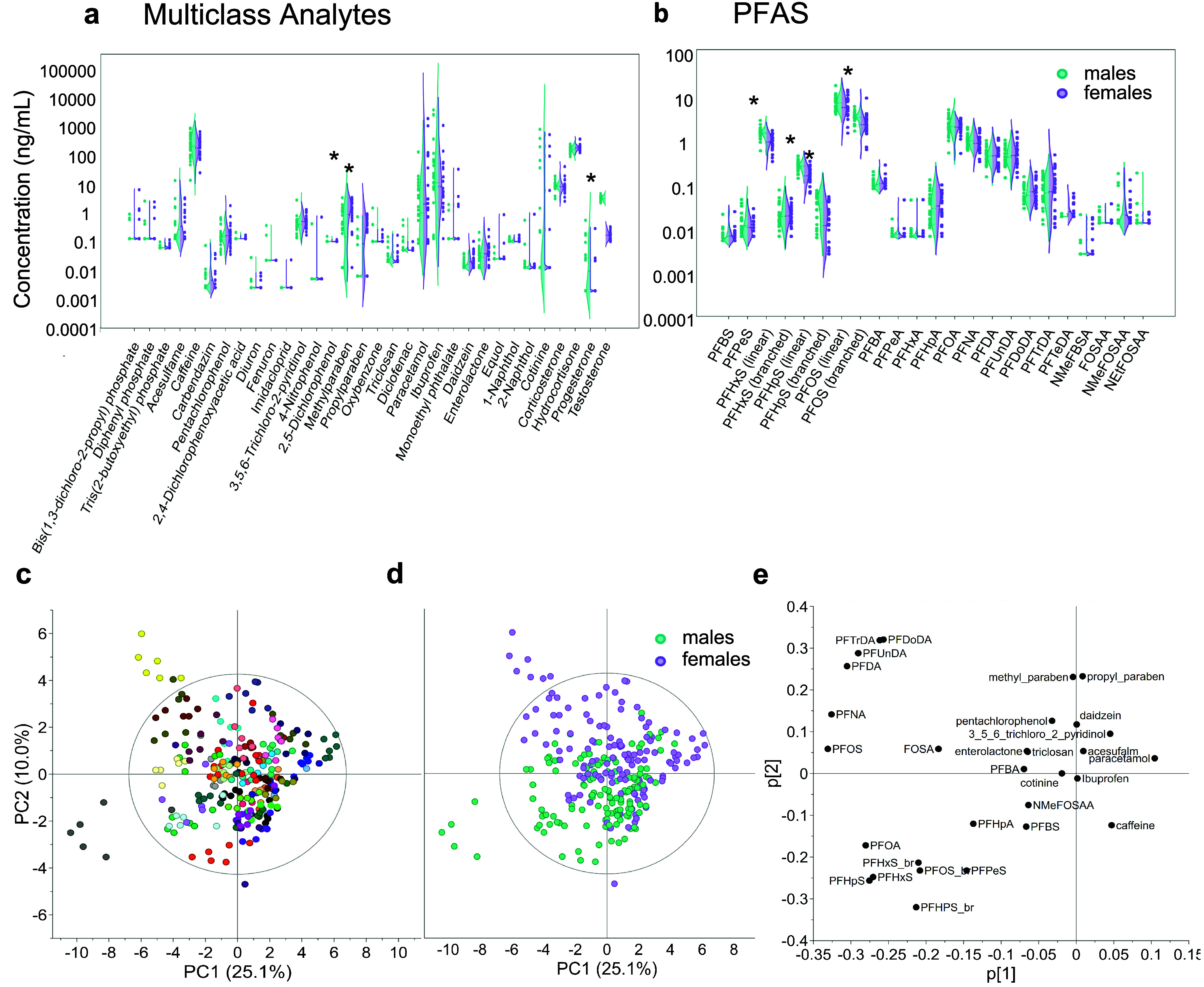
Targeted chemical exposome and individual variation in S3WP participants. (a, b) Violin plots of average analyte concentrations per individual (log_10_ scale) for targeted multiclass analytes and targeted perfluoroalkyl and polyfluoroalkyl substances (PFAS), respectively, with color coding according to males (green, *n* = 23) and females (purple, *n* = 23). Dashed lines on violin plots indicate mean concentration for each analyte in each sex. Asterisks on top of violin plots indicate the analytes with a significantly different concentration between males and females (Bonferroni corrected *p* < 0.01; Wilcoxon signed-rank test, *n* = 138 for each sex). (c, d) Six sampling visits of each individual shown in principal component analysis (PCA) scores plots (3 components; R2Xcum = 43.4%, Q2cum = 29.8%) with samples color-coded by participant ID (c) and sex (d). (e) PCA loadings of the targeted analytes. In the PCA plots, only analytes with a detection frequency >10% are shown, and excluding steroid hormones.

Among all plasma samples, 57 of the 83 target analytes were detected and quantified. These substances belonged to 14 diverse chemical classes (Table S3 and Figure 1a,b), including pesticides (organophosphate and neonicotinoid), flame retardants, PFAS, personal care products, pharmaceuticals, plasticizers, dietary substances, polycyclic aromatic compounds, a nicotine metabolite, and endogenous steroid hormones. For statistical and multivariate analysis, only target analytes with DF > 10% were considered (34 analytes from 9 chemical classes, Figure S1). Comparing mean plasma concentrations of males (*n* = 138) and females (*n* = 138), significant differences by sex were identified for parabens as well as certain PFAS and hormones (Figure 1a,b). These were identified with Wilcoxon signed-rank tests (Bonferroni corrected *p*-values), which were applied after showing non-normal distributions for all 34 analytes (Shapiro-Wilk test, *p* < 0.05). Methylparaben and propylparaben, which are used in cosmetics, had significantly higher levels in females (methylparaben: males 0.60 ng/mL, females 1.5 ng/mL, *p* < 0.001; propylparaben: males 0.06 ng/mL, females 0.31 ng/mL, *p* < 0.01, Figure 1a), consistent with a previous study in urine.^30^ Conversely, females had significantly lower concentrations of several PFAS (Figure 1b), consistent with known routes of PFAS elimination during pregnancy, breastfeeding, and menstruation,^31−34^ including for linear PFHxS (males 1.4 ng/mL, females 0.90 ng/mL, *p* < 0.001), linear PFHpS (males 0.25 ng/mL, females 0.16 ng/mL, *p* < 0.001), branched PFHpS (males 0.033 ng/mL, females 0.017 ng/mL, *p* < 0.001) and branched PFOS (males 3.11 ng/mL, females 2.40 ng/mL, *p* < 0.001). As expected, mean testosterone was significantly higher in males (3.2 ng/mL) than females (0.18 ng/mL, *p* < 0.001) (Figure 1a), with levels in reference ranges for healthy adults in comparable age groups.^35^

After excluding the detected sex hormones (hydrocortisone, corticosterone, progesterone, and testosterone), PCA was applied to broadly visualize and detect sources of intra- and interindividual variability of environmental exposures (30 targets, Figure 1c–e). In the PCA scores plot colored by S3WP participant ID (Figure 1c), the 6 samples from each individual were often grouped together, indicating relative stability of the target exposome over 2 years. Notably, two individuals were consistently outliers relative to the study population, showing particularly high levels of certain PFAS (i.e., C10–C13 perfluoroalkyl carboxylates for female W0043 colored in yellow; C5–C8 perfluoroalkyl sulfonates for male W0074, colored in gray, Figure 1e). Partial separation between males and females along PC2 (10% of variation, Figure 1d) was mostly explained by sex-specific exposures to parabens and PFAS (Figure 1e), consistent with the statistical differences noted above.

### Untargeted Molecular Discovery

Among all 276 plasma samples, a total of 129,547 unique untargeted features were detected across ESI+ and ESI– after blank filtering and removal of redundant features; thus, targeted analytes represented only 0.04% of the overall molecular data set. Substantially fewer features were detectable in any individual (mean 73,426 features per individual, range 67,405–87,159 features, Figure S2). Moreover, only 20,520 features (16% of total data set) were detected in all individuals in at least one visit, somewhat lower than for the targeted analytes (i.e., 26% of targets were detected in all individuals for at least 1 time point). These results indicate that a high proportion of “molecular dark matter”^36^ in plasma is unique to individuals, possibly representing unique environmental exposures, and/or unique biological response at the metabolome level. This finding is consistent with a recent observation that gut microbiota composition (i.e., internal exposome) explains the majority of variance (i.e., 58%) in individual human plasma metabolites,^37^ although other sources of environmental exposure have yet to be similarly examined. In untargeted studies of the exposome or the metabolome, it is therefore predictable that the complexity of molecular data sets will increase with larger sample sizes.

By automated matching to open access libraries (based on MS1 accurate mass and MS2 spectra) and after manual curation of all annotations, a total of 462 high-confidence structural candidates were assigned (i.e., at least Level 2a confidence^38^) in ESI+ and ESI–, corresponding to a 0.4% annotation rate; this total does not include the targeted analytes, which are considered confirmed at Level 1. Combining all Level 1 identified chemicals (targeted analytes and untargeted discoveries confirmed by reference standard;^38^ see next section), and all Level 2 high-confidence annotations, resulted in a total of 519 substances, including 343 environmental chemicals, 162 endogenous metabolites, and 14 substances with ambiguous classification (Table S4). The 343 environmental chemicals were divided into 11 subclasses, including dietary substances, drugs, industrial chemicals, PFAS, plasticizers, and personal care products (Figure S3), and are hereafter referred to as the “chemical exposome”. The 162 endogenous metabolites were divided into 7 subclasses, including fatty acids, bile acids, amino acids, and hormones. We acknowledge that a strict separation between chemical exposome and endogenous substances leaves certain ambiguities, including for the reason that the human metabolome includes the endogenous metabolomes of other species consumed through diet.^39^

### Confirmation of Selected Structural Annotations

To confirm the Level 2 untargeted molecular annotations, authentic standards (>98% purity) were obtained for 25 analytes, including 3 endogenous and 22 environmental substances. Confirmations were successful for 20 analytes (80% success rate, Tables S5, S6 and Figures S4, S21), indicating an effective untargeted acquisition and data-processing workflow for molecular discovery. When a confirmed analyte was also detectable in the pooled Swedish reference plasma, and its concentration could be quantified with a standard addition curve (0–10 ng/mL range), reference standardization^13^ was used for semiquantification in individual samples (Table S5). Selected examples of confirmed molecular discoveries, including unexpected and widespread environmental exposures, are discussed below.

#### Rubber Additives

The substance 1,3-diphenyl guanidine (Table S5 and Figure S4) was recently reported to be the most frequently detected tire-derived contaminant in air^40^ and indoor dust^41^ globally and was confirmed here in 83% of samples. Reports of 1,3-diphenyl guanidine in human biofluids remain rare.^42−44^ A related substance, 4-*tert*-butylcatechol (TBC, Table S5 and Figure S7), was detected in 92% of samples, despite no previous reports in human biofluids. This substance is reported as a contact allergen,^45^ and is used as an additive polymerization inhibitor in the rubber, paint, and petroleum industry.^45,46^

#### Industrial Chemicals

Triphenylphosphine oxide (TPPO) is a widely used synthetic intermediate in pharmaceutical products^47^ and was detected here in 3% of samples (Table S5 and Figure S5). TPPO has begun to be widely detected in indoor air, dust, and aquatic systems,^48,49^ but has only rarely been detected in human samples.^42,47^ The isomers 1- and 2-naphthalenesulfonate (Table S5 and Figure S12), used in textile, pharmaceutical, and agrochemical production,^50,51^ were confirmed in 13% of samples here. These two substances have low biodegradability^50,51^ and neither has been previously confirmed in human biofluids (2-naphthalenesulfonate has been reported previously at Level 2^7^).

We also confirmed 2,6-di*tert*-butyl-4-nitrophenol (DBNP, Table S5 and Figure S6), a known transformation product of 2,6-di*tert*-butyl-4-methylphenol, a widely used synthetic antioxidant added to polymers, foods, and cosmetics.^52^ DBNP has been reported in the environment^53,54^ and in plastic food packaging^55^ but not in human biofluids. Its detection in 20% of Swedish samples here deserves further attention due to its biological persistence and toxic potential.^54^ Two benzotriazole isomers, 4-methyl- and 5-methyl-1H-benzotriazole, were also confirmed and semiquantified in 9% of samples (Table S5 and Figure S10). These benzotriazoles are high-production volume chemicals mainly applied as corrosion inhibitors and ultraviolet stabilizers, widely detected in environmental matrices^56−58^ but also in a few studies in human samples.^59,60^

#### Pesticides

Chlorothalonil-4-hydroxy was confirmed and semiquantified in 100% of plasma samples (1.1–11.5 ng/mL, median 3.7 ng/mL; Table S5 and Figure S9). This finding is consistent with a targeted study of pregnant Swedish women (1997–2015) where it was also detected in all samples (median 4.1 ng/mL),^61^ yet it remains rarely monitored in humans. It is a known transformation product of the fungicide chlorothalonil, which has not been permitted for agricultural use in Sweden since the 1990s,^62^ and has been banned in the EU since 2019 due to its carcinogenic properties and risk to fish and amphibians.^63^ Chlorothalonil-4-hydroxy is considered more toxic,^64^ more persistent, and more mobile in soil^65^ than the parent pesticide; thus, its widespread presence in blood deserves attention in exposome studies.

#### Personal Care Products

Sodium lauryl sulfate was confirmed in 45% of samples (Table S5 and Figure S8), and has not previously been reported in human biofluids to our knowledge, despite being a major surfactant in shampoos.^66,67^ It is known to be absorbed into the bloodstream in animal models,^68^ and may also be inhalable during shampoo use.^69^ This surfactant is rarely monitored and remains unregulated due to its biodegradable nature, but its risks have been debated.^70^

### Longitudinal Stability of the Chemical Exposome

The final data set of 519 annotated substances (Levels 1 and 2) was used to examine the intra- and interindividual exposure variation in this 2 year study with six sampling points. For each substance, we calculated the ICC (Table S7), a nondimensional ratio of the interindividual variance to the total variance (i.e., the sum of inter- and intraindividual variance).^71^ In the context of exposome research, ICCs are instructive for study design as they describe the extent to which individuals retain their rank order in a study population with repeated measurements of exposure over time.^72^ Higher ICCs correspond to more stable exposures, that can therefore be measured fewer times throughout the life course, whereas lower ICCs correspond to less stable exposures that may need several repeated measurements to accurately classify exposure over the life course. ICCs range from 0 to 1, with values <0.40 corresponding to poor reproducibility of repeated measurements, 0.40 to 0.75 is considered fair to good, and >0.75 indicates excellent reproducibility.^71^

**Figure 2:**
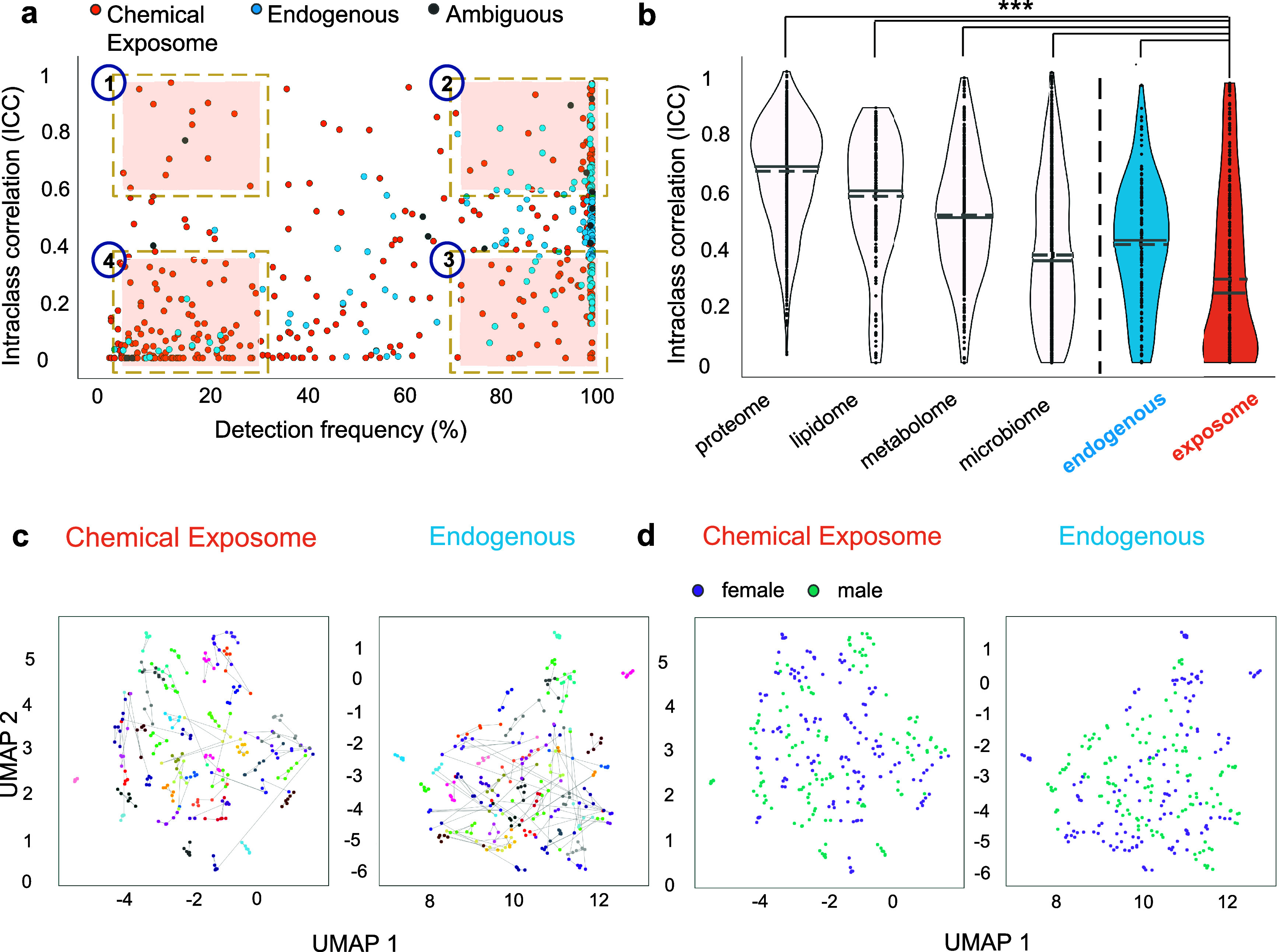
Longitudinal stability of the chemical exposome and comparison to other molecular profiles. (a) Calculated intraclass correlation coefficients (ICC) of the 519 annotated substances (Level 1 and Level 2) along with their detection frequency (DF), for the chemical exposome (orange, *n* = 343), endogenous metabolites (blue, *n* = 162) and compounds with ambiguous origin (gray, *n* = 14). Four regions of interest are highlighted on the plot: top left (Type 1; rare-stable, low DF, high ICC), top right (Type 2; common-stable, high DF, high ICC), bottom right (Type 3; common-unstable, high DF, low ICC), bottom left (Type 4; rare-unstable, low DF, low ICC). (b) ICC data presented in violin plots separately for the chemical exposome and the endogenous metabolites as well as for the proteome, lipidome, metabolome, and microbiome from Tebani et al.,^11^ including only individuals present in the exposome study. The solid and dashed lines show median and mean ICC values, respectively, and asterisks indicate significantly different ICC means between the chemical exposome and endogenous metabolites (*p* < 0.001; Student’s *t* test, two-tailed) and between the chemical exposome and all other molecular profiles (*p* < 0.001; one-way ANOVA and Tukey’s test). (c, d) Global profiles across visits applying uniform manifold approximation and projection (UMAP) for the chemical exposome and the endogenous metabolites color-coded by participant ID with gray lines connecting samples representing different visits of the same individual (c) or color-coded by sex (d).

The majority of annotated substances in plasma (306 of 519 analytes) had ICCs < 0.40 (Figure 2a), and the mean ICC was significantly higher for endogenous metabolites (0.40) than for the chemical exposome (0.30, Student’s *t* test, two-tailed, *p* < 0.001) (Figure 2b). Moreover, while the ICCs for endogenous metabolites were normally distributed, the chemical exposome showed denser distributions toward lower and higher ICC values. More specifically, 66% of ICCs were <0.4 for the chemical exposome, compared to only 45% of ICCs for endogenous metabolites, and 10% of the ICCs were >0.75 for the chemical exposome, compared to only 6% of ICCs for endogenous metabolites (Figure 2b). While the plasma chemical exposome and metabolome have highly stable components, these results mean that the majority of small molecules in plasma must be measured more than once over time to adequately represent an individual’s exposure. A similar conclusion was reached for the target analysis of 24 nonpersistent environmental chemicals in urine of pregnant women and children.^73^

To conclusively link environmental exposures to disease etiology, an objective in exposomics is to identify key perturbations in molecular networks at the metabolome, proteome, and gene expression levels.^74^ In longitudinal exposome studies, this might be addressed through multiomics, but the relative dynamics of each molecular data set must be considered at the study design stages. Thus, here we compare our results to those of Tebani et al., who reported ICCs for several multiomic profiles in the same study participants.^11^ We recalculated the ICCs of Tebani et al. to only include individuals with paired chemical exposome measurements (see the Supporting Information), and compared to the relatively low stability of the chemical exposome (mean ICC 0.30, median 0.23), significantly higher mean ICCs were evident for the plasma proteome (mean 0.65, median 0.67), lipidome (mean 0.55, median 0.59), metabolome (mean 0.50, median 0.50), and gut microbiota (mean 0.38, median 0.35) (Figure 2b; one-way ANOVA, Tukey’s test *p* < 0.001). These results emphasize the importance of repeated measures of the chemical exposome in epidemiological studies to minimize exposure misclassification, and in longitudinal multiomic studies, the exposome should be measured as frequently, or more frequently, than other biomolecular profiles.

The gut microbiome had the most similar distribution of ICCs in comparison to the chemical exposome, with overall lower reproducibility and similar high density at lowest values (Figure 2b). Like the chemical exposome, gut microbiota is shaped by life course exposures, including diet, disease history, and medication, as well as by intrinsic factors such as age and host genetic variation.^37^ As discussed above, the known link between gut microbiota and plasma small molecules^37^ could partially explain similar ICC distributions for gut microbiome and the plasma chemical exposome.

UMAP analysis allowed the relative variation of individual exposomes over time to be visualized relative to the study population (Figure 2c). The visits of each individual, colored by participant ID and connected by lines, demonstrate that the complex chemical exposome profile of most individuals has stable factors over time, despite the overall low temporal stability described above (Figure 2c). However, individual variability was evident for both the chemical exposome and endogenous metabolites, with some individuals having remarkably unique and stable profiles and others displaying higher variability between visits (Figure 2c). UMAP visualization of metabolome stability in Tebani et al.^11^ showed similar results. Notably, UMAP projections of the chemical exposome and endogenous metabolites did not indicate any sex differences (Figure 2d).

### Longitudinal Exposure Types

A plot of each chemical’s ICC versus sample detection frequency (Figure 2a) allowed for new insights into chemical exposure characterization that would not have been evident without repeated sampling. Four exposure types (insets#1–4, Figure 2a; see Table S7 for the categorization of each compound) are defined and discussed in this context. First, we categorize Type 1 exposures as rare-stable (i.e., approximately DF < 20%, ICC 0.6–1, 2% of the data set). These exposures may be of high relevance to the small fraction of individuals with consistently elevated exposure over the life course, but could be easily overlooked in cross-sectional study designs, due to being rare in the population. These exposures are therefore of high relevance in a precision health context, as they likely arise from unique behaviors, occupation, or lifestyle factors that could be mitigated if identified. An example was the industrial intermediate triphenylphosphine oxide (ICC 0.64, 3.2% DF; Level 1) which was only detected in 9 samples overall, but consistently in all 6 samples of individual W0015. Other examples included NEtFOSAA (ICC 0.93, 7% DF; Level 1) (consistent for individual W0008), a PFOS precursor with exposure sources linked to food packaging^75^ and the drug citalopram (ICC 0.88, 9% DF; Level 2)^75^ (consistent for individual W0090), which acts as a selective serotonin reuptake inhibitor.^76^

Type 2 exposures were categorized as common-stable (i.e., approximately DF > 80%, ICC 0.6–1, 12% of the data set) and include exposures that are widespread in the population, and with stable levels in individuals. A large proportion of these substances were PFAS and chlorinated analytes, which have biological persistence and long pharmacokinetic half-lives. For example, PFHxS had an ICC of 0.94 (100% DF, reported half-life 8.5 yrs)^77^ and PFOS isomers had ICCs of 0.95 (100% DF, reported half-life 5.4 yrs for linear PFOS).^77^ Another example was the fungicide transformation product chlorothalonil-4-hydroxy (ICC 0.80, 100% DF; confirmed Level 1). The half-life of chlorothalonil-4-hydroxy in humans has not yet been determined, but the high ICC reported here and in a previous study of Costa Rican women (ICC 0.81)^61^ emphasizes the importance of further studies for this environmentally persistent chemical. Other Type 2 substances nevertheless included analytes with reported fast elimination half-lives in the range of days or hours. Such examples were 3-hydroxycotinine (ICC 0.91, 88% DF, half-life 6.6 h;^78^ confirmed Level 1), and the pesticide pentachlorophenol (ICC 0.83, 98% DF, half-life 20 d;^79^ Level 1). These latter results suggest that, despite relatively rapid elimination pharmacokinetics, consistent lifestyle factors over the course of 2 years (e.g., tobacco use) can result in steady levels of environmental substances in plasma. In fact, the above ICCs were comparable to that of the targeted steroid hormone testosterone (ICC 0.95, 100% DF; Level 1). While substances in the Type 2 category do not necessarily require repeated sampling to accurately classify exposure in health studies, longitudinal studies with untargeted chemical exposomics may be a powerful approach to discovering emerging persistent chemicals in the population. For example, among the thousands of nonannotated molecular features detected here, these could be prioritized for identification based on simultaneously high ICC and DF.

Type 3 exposures were categorized as common-unstable (i.e., approximately DF > 80%, ICC < 0.4, 18% of the data set). Substances in this category are widespread in the population, but have unstable levels in individuals; thus, the health relevance of these can most powerfully be explored in longitudinal studies with repeated measurements. Examples here included substances associated with exposure through diet, and/or having short elimination half-lives, such as the insecticide metabolite 3,5,6-trichloro-pyridinol (ICC 0.25, 97% DF, half-life 27 h;^80^ Level 1), which is linked to consumption of imported foods in Sweden.^81^ Other examples are the caffeine metabolite theobromine (ICC 0.31, 100% DF, half-life 7–12 h;^82^ Level 2), azelaic acid which is found in grains^83^ (ICC 0.40, 100% DF; confirmed Level 1), the herbicide ioxynil^84^ (ICC 0.37, 92% DF; Level 2) and the above-discussed rubber additive 1,3-diphenyl guanidine (ICC < 0.01, 83% DF, half-life 9.6 h in rats;^41^ Level 1).

Finally, Type 4 exposures were categorized as rare-unstable (i.e., approximately DF < 20%, ICC < 0.4, 23% of the data set). These exposures are more difficult to understand with regard to health and could be deprioritized in exposome studies to further reduce the dimensional complexity and multiple testing. Examples of Type 4 exposures are the industrial chemicals 2,6-di*tert*-butyl-4-nitrophenol (ICC 0.33, 20% DF; Level 1) and the isomers 1- and 2-naphthalenesulfonate (ICC 0.29, 13% DF; Level 1), both discussed above.

### Correlated Coexposures

**Figure 3:**
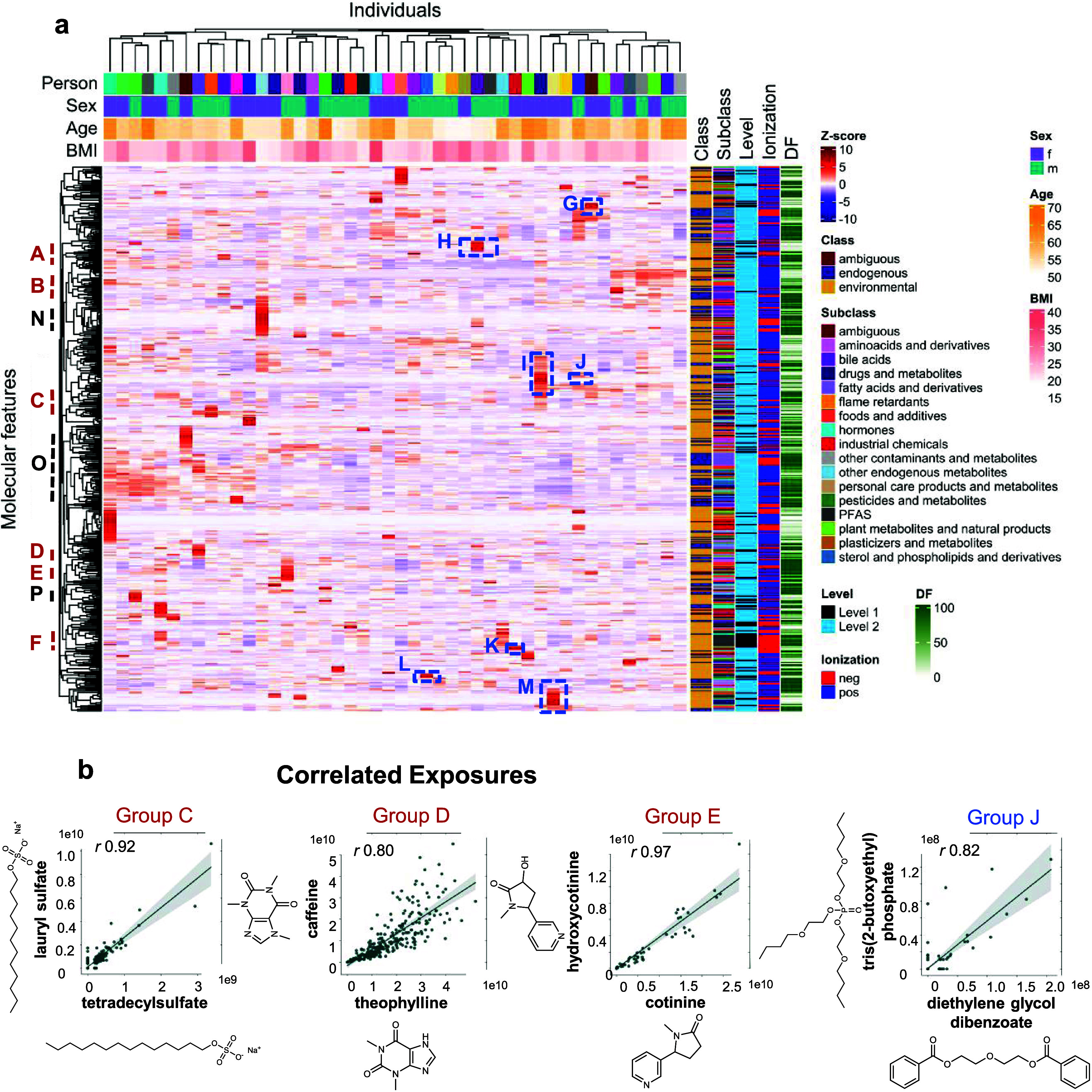
Clustered exposome profiles of S3WP participants and examples of correlated exposures. (a) Hierarchical cluster analysis heatmap with dendrograms showing the exposome profiles of 46 individuals, each averaged across the 6 clinical visits for 519 annotated substances (Level 1 and Level 2). Color coding of chemical substances (“molecular features”) is according to class, subclass, confidence level of identification, ionization, and detection frequency (DF). Color coding of individuals is according to sex, age, and BMI. Groups of interest are highlighted on the heatmap (in red A–F: common coexposures; in blue G–M: rare coexposures; in black N–P: endogenous metabolites, see zoomed-in versions in Figures S23, S24, and S27). Common coexposures were detected in many participants while rare coexposures were detected only in individuals or a small population fraction. (b) Linear regression plots with 95% confidence intervals across all 6 visits (*n* = 276) between features that clustered together in the heatmap. The Pearson correlation coefficient (*r*) is shown for each regression and all analyte pairs showed a significant correlation (*p*-value <0.001).

The complexity and high dimensionality of the chemical exposome represent major challenges to its implementation in health studies; however, it has been proposed that reduction of its dimensional complexity should be possible by grouping correlated exposures.^74^ Such coexposures may arise if two or more substances have common exposure sources and similar pharmacokinetics, resulting in correlated dynamics. Here we highlight several coexposures among the 46 participants that were evident in HCA heatmaps (Figures 3a,and S22), and for discussion we group these into “common” and “rare” coexposures based on whether these were evident among many participants (Figure 3a, groups A–F, zoom-in Figure S23), or only in individuals or a small fraction of the population (Figure 3a, groups G–M, zoom-in Figure S24), respectively. Both HCA heatmaps are based on all 46 participants and 519 annotated substances (Levels 1 and 2), but for simplicity, we focus on Figure 3a, which is based on the average response of each substance for each individual’s six visits. Figure S22 shows the chemical exposome at each visit to simultaneously visualize intra- and interindividual patterns. To guide interpretation and hypothesis generation, both heatmaps were made interactive (available at https://s3wp-exposomics.serve.scilifelab.se) and metadata are displayed for each substance (i.e., chemical class, subclass, confidence level, ionization mode, DF) and participant (sex, age, BMI).

One common coexposure (Group A, Figure 3a, zoom-in Figure S23) included the targeted fungicide pentachlorophenol (ICC 0.83, 98% DF) and the dietary fatty acid, pentadecanoic acid, which is a biomarker of dairy consumption^85^ (Level 2; ICC 0.29, 93% DF; *r* 0.43, *p* < 0.005). Pentachlorophenol is also a metabolite of the organochlorine pesticide hexachlorobenzene^86^ which has been associated with dairy consumption in Sweden.^87^ Another common coexposure (Group B, Figure S23) included homologues of poly(ethylene glycol) (Level 2, ICC 0–0.31, 36–100% DF), which are approved by the US Food and Drug Administration^88^ and have been previously detected in environmental,^89,90^ but not in human samples. Group C coexposures (Figure S23) included sodium lauryl sulfate (ICC 0.09, 45% DF, confirmed Level 1), strongly correlated with tetradecyl sulfate (Level 2; ICC 0.07, 36% DF; *r* 0.92, *p* < 0.001, *n* = 276, Figure 3b) and lauryl diethanolamide (Level 2; ICC 0.01, 32% DF; *r* 0.66, *p* < 0.001, *n* = 276), all of which are ingredients in household cleaning and personal care products.^66,67,91^ Such correlations between confirmed analytes (targeted/Level 1) and one or more Level 2 substances in a related chemical class provide further confidence in the Level 2 annotations, and more examples are discussed below.

**Figure 4:**
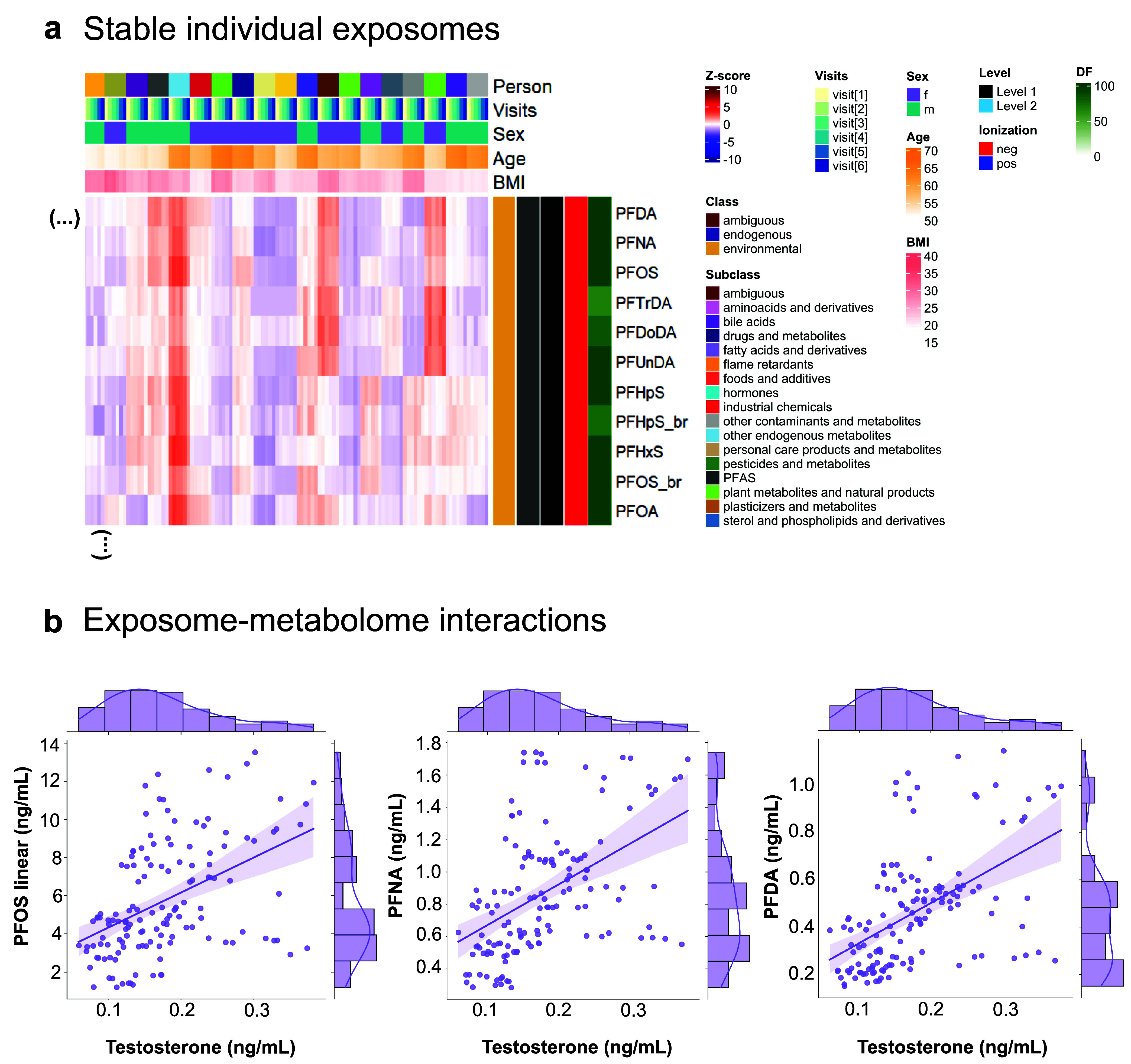
Stable PFAS exposome profiles and examples of exposome-metabolome interactions. (a) Zoomed-in subset of the hierarchical cluster analysis heatmap (19 out of 46 participants) with 6 visits shown per individual where a stable group of PFAS is observed. Color coding of features is according to class, subclass, confidence level of identification, ionization, and detection frequency (DF). Color coding of individuals is according to sex, age, and BMI. (b) Linear regression plots with 95% confidence intervals between testosterone and PFAS targets for female individuals across all 6 visits (*n* = 138). All analyte pairs showed significant correlation (*p*-value <0.001). The plots also show the analyte distributions in histograms.

In Group D (Figure S23), the targeted analyte caffeine (ICC 0.50, 99% DF) correlated strongly with the Level 2 caffeine metabolites^92^ theobromine (ICC 0.31, 99.6% DF; *r* 0.60, *p* < 0.001, *n* = 276 individual samples) and theophylline (ICC 0.52, 100% DF; *r* 0.80, *p* < 0.001, *n* = 276, Figure 3b). While these three alkaloids are typical of coffee plants, they also clustered with 4-methylcatechol (Level 2; ICC 0.60, 99% DF), a compound generated during the roasting of coffee beans.^93^ Similarly, in Group E (Figure S23) the targeted nicotine metabolite, cotinine (ICC 0.94, 20% DF), was strongly correlated with a secondary nicotine metabolite, 3-hydroxycotinine^94^ (ICC 0.91, 88% DF; *r* 0.97, *p* < 0.001, *n* = 276, Figure 3b), confirmed here at Level 1. Group F (Figure S23) shows the prominent PFAS class as a coexposure cluster (i.e., many PFAS were highly correlated, Figure S25). Additionally, the HCA heatmap with all 6 visits (Figure S22) provides visual evidence for the high ICCs of these substances, whereby the majority of individuals showed consistently high (red) or low (blue) responses across all visits (also see zoom-in subset in Figure 4a).

Examples of rare coexposures involved groups of chemicals occurring at low DF in the population (approximately <20%), but also include distinctively higher levels of more commonly detected substances in individuals. In Group G (Figure 3a), one distinctive individual had multiple rare coexposures (zoom-in Figure S24), including triphenylphosphine oxide (ICC 0.64, 3.2% DF; Level 1) which was detected in all 6 samples of this individual. As noted above, this substance is used as an intermediate in pharmaceutical products;^47^ thus, it was interesting to note correlated coexposures in this individual to 2 drugs, trimethoprim^95^ (Level 2, ICC < 0.01, 14% DF) and ketoprofen^96^ (Level 2, ICC 0.34, 57% DF), as well as to the targeted flame-retardant, bis(1,3-dichloro-2-propyl) phosphate (ICC < 0.01, 12% DF) and the long-chain PFAS, perfluorotetradecanoate (PFTeDA; ICC 0.31, 9% DF). The reasons for these shared rare exposures are not currently understood, but deserve follow-up study, and would not have been uncovered without multiclass targeted and untargeted chemical exposomics.

In Group J (Figure S24), two rare exposures, namely, the targeted flame-retardant and plasticizer tris(2-butoxyethyl) phosphate (ICC 0.01, 9% DF) and the plasticizer diethylene glycol dibenzoate (Level 2; ICC < 0.01, 9% DF), were strongly correlated (*r* 0.82, *p* < 0.001, *n* = 276, Figure 3b). In another rare coexposure (Group K, Figure S24) the targeted personal care product triclosan (ICC 0.45, 16% DF) clustered with several other chlorinated substances, such as the herbicide 4-chlorophenoxyacetic acid^97^ (Level 2; ICC 0.50, 48% DF), the pesticide chloroxynil^98^ (Level 2; ICC 0.01, 33% DF), and an isomer of chlorophenol (Level 2; ICC 0.16, 2.2% DF), the latter discovered based on exact mass, chlorine isotopic pattern (Figure S11) and RT adjacent to 4-chlorophenol (confirmed Level 1). In Group L, the targeted herbicide diuron (ICC < 0.01, 20% DF) clustered with the targeted fungicide carbendazim (ICC < 0.01, 4% DF) and the herbicide ioxynil^84^ (Level 2; ICC 0.37, 92% DF) (Figure S24).

### Chemical Exposomics as a Tool for Precision Health

An individual’s drug metabolism capacity is influenced by both genetic and environmental factors, and understanding these factors is a key objective of personalized medicine, for example, toward precision dosing of drugs.^99^ We hypothesized that untargeted chemical exposomics could be a useful tool for monitoring drug efficacy (or toxicity) at the individual level by monitoring drugs and detecting their known or unknown metabolites in plasma. We first observed that groups of rare coexposures often consisted of drugs and their known metabolites (Figure S24). For instance, the anti-inflammatory drug diclofenac (Level 1; ICC 0.19, 6.9% DF) and 5-hydroxy diclofenac^100^ (Level 2; ICC 0.37, 9.8% DF) in Group H, the antihistamine cetirizine^101^ (Level 2; ICC 0.88, 21% DF) and hydroxyzine (Level 2; ICC0.83, 68% DF) in Group I, and the antidepressant sertraline (Level 2; ICC 0.32, 2.9% DF) and norsertraline^102^ (Level 2; ICC 0.33, 2.5% DF) in Group M. To further explore the potential of chemical exposomics to identify drug metabolites whose MS2 spectra are unknown, or not publicly available, we suspect-screened all 38,499 nonannotated untargeted features detected in ESI- for: (i) theoretical *m*/*z* corresponding to hydroxyl, sulfate or glucuronide metabolites of the above-mentioned pairs of parent drugs and metabolites, and (ii) correlation with the respective parent drug or metabolites (i.e., *p* < 0.001, *n* = 276 individual samples, Figure S26). By these combined criteria, a suspected additional hydroxy metabolite of diclofenac (*m*/*z* 310.0049, RT 10.74 min, 2 ppm error, ESI-) was found that correlated both with diclofenac (*r* = 0.61, *m*/*z* 294.0094, RT 13.16 min, ESI-) and 5-hydroxy diclofenac (*r* = 0.92, *m*/*z* 312.0190, RT 11.42 min, ESI+), and the corresponding suspect sulfate conjugate (*m*/*z* 389.9616, RT 9.50 min, 1.3 ppm error, ESI-) correlated moderately with diclofenac (*r* = 0.63), and strongly with the new hydroxy metabolite (*r* = 0.98). Finally, a suspected glucuronide of sertraline (sertraline carbamoyl-*O*-glucuronide, *m*/*z* 524.0884, RT 13.75 min, 0.1 ppm error, ESI-) correlated strongly with both sertraline (*r* = 0.97, *m*/*z* 306.0812, RT 14.09 min, ESI+) and norsertraline (*r* = 0.98, *m*/*z* 292.0653, RT 14.31 min, ESI+). These results demonstrate that untargeted chemical exposomics is a feasible tool to inform the pharmaceutical treatment of individuals.

Hierarchical clustering of natural compounds and endogenous metabolites (Level 2, Figure 3a, groups N–P, zoom-in Figure S27) was also observed. These may be useful indicators of behavior (e.g., dietary habits) or biomarkers of disorders or disease risk. For example, in group N, a single individual deviated from the rest of the population due to the high relative response of several bile acids, including cholic, glycochenodeoxycholic, and ursocholic acid (Figures S27 and S28a), which can indicate hepatic impairment.^103^ Moreover, 12 participants clustered with relatively high responses of several lipids, including the fatty acids cis-4,10,13,16-docosatetraenoic acid (ICC 0.55, 100% DF), 9Z, 12Z-linoleic acid (ICC 0.44, 100% DF), and arachidonic acid (ICC 0.42, 100% DF), with high correlations among these (Group O, Figures S27 and S28b). High correlations were observed between the microbial metabolite phenylacetylglutamine (ICC 0.65, 100% DF) and the two uremic toxins p-cresol sulfate (ICC 0.62, 100% DF; *r* 0.80, *p* < 0.001, *n* = 276) and 3-indoxyl sulfate (ICC 0.51, 100% DF; *r* 0.49, *p* < 0.001, *n* = 276) (Group P, Figures S27 and S28c). This microbial metabolite and the associated uremic solutes accumulate in patients with chronic kidney disease, and their levels have been associated with adverse outcomes including cardiovascular disease.^37,104^

### Exposome-Metabolome Interaction

Correlations between environmental chemicals and endogenous metabolites can be suggestive of exposome-metabolome interactions, whereby exposure(s) induces a metabolic response. Although the current study is relatively small, we examined for evidence of endocrine disruption between testosterone and PFAS, which are targeted analytes that were detected in most samples. Testosterone and PFAS did not cluster together in the HCA heatmaps because men have much higher testosterone levels than women, and any correlations may be sex-specific. Among all females, positive correlations were observed between testosterone and several PFAS (Figure 4b), including linear PFOS, perfluorononanoate (PFNA), and perfluorodecanoate (PFDA). These three associations were statistically significant in mixed-effect models, which account for the repeated sampling of individuals, and PFOS and PFDA remained significant in adjusted models controlling for baseline age and BMI (Table S8; *p* < 0.02 after Bonferroni correction). Positive associations between PFOS, PFOA, and PFHxS with total and free testosterone have been reported in postmenopausal women (median 63 yrs),^105^ similar to the age of women here (median 57 yrs). However, the same study reported no similar effect for PFNA and PFDA on any reproductive hormones.^105^ These findings for PFAS deserve further study considering that a meta-analysis of prospective studies has reported that higher blood testosterone was associated with an increased risk of breast cancer in postmenopausal women.^106^

### Advances, Limitations, and Future Directions

This study represents the first application of longitudinal chemical exposome profiling in the human blood of multiple individuals. The combined targeted and untargeted chemical exposomics workflow enabled sensitive and precise quantification of priority targeted analytes with simultaneous potential to discover unexpected exposures of potential health relevance. In this longitudinal study of only 46 individuals, we also report unique coexposures, and statistically significant exposome-metabolome interactions, demonstrating promise for chemical exposomics in precision health applications.^107^ Nevertheless, the vast majority of untargeted molecular features remain unidentified, and the underlying laboratory methods and data analyses must be scaled up for larger studies. While it has been envisioned that unraveling the effects of the chemical exposome on the progression of disease will require network science and systems biology approaches to integrate chemical and biomolecular interactions across many omic levels,^74^ the relatively low stability of the chemical exposome stands as a major practical challenge to this approach. The wide range of ICCs for hundreds of endogenous and exogenous small molecules reported here can therefore assist in the design of future longitudinal cohorts focused on the chemical exposome.

## Data Availability

Interactive HCA heatmaps are available at https://s3wp-exposomics.serve.scilifelab.se. Access to the MS data and participant-level data sets will be made available upon reasonable request by contacting the corresponding author

## Supporting Information Available

Methods; detected target analytes (Figure S1); number of annotated features (Figure S2); total number of targeted and annotated untargeted analytes (Figure S3); extracted ion chromatogram and DDA spectrum (Figures S4–S21); hierarchical cluster analysis heatmap (Figure S22); zoomed-in sections of the hierarchical cluster analysis heatmap (Figures S23 and S24); correlations observed for PFAS targeted analytes (Figure S25); correlations observed for Level 1 and Level 2 drugs and drug metabolites (Figure S26); zoomed-in sections of hierarchical cluster analysis heatmap (Figure S27); correlations observed for Level 2 analytes (Figure S28); correlation observed for BADGE-H_2_O annotated at Level 2 (Figure S29); untargeted molecular discoveries (Table S5)(PDF)
Targeted analytes, method validation, and corresponding native standards (Table S1); MS-DIAL parameters (Table S2); detected and quantified targeted analytes (Table S3); Levels 1 and 2 annotated substances (Table S4); analytes selected for confirmation (Table S6); intraclass correlation coefficient (Table S7); and mixed-effect model parameters (Table S8) (XLSX)
