# Supplementary material for: Longitudinal Exposomics in a Multiomic Wellness Cohort Reveals Distinctive and Dynamic Environmental Chemical Mixtures in Blood
Source: Environ Sci Technol. 2024 Sep 5;58(37):16302–15. doi: 10.1021/acs.est.4c05235 (PMC11411717; doi:10.1021/acs.est.4c05235)
Supplement: Supplementary file 1 — es4c05235_si_001.pdf [file es4c05235_si_001.pdf]

Longitudinal Exposomics in a Multiomic Wellness Cohort Reveals Distinctive and Dynamic Environmental Chemical Mixtures in Blood

Kalliroi Sdougkou[1], Stefano Papazian[1,2], Bénilde Bonnefille[1,2], Hongyu Xie[1], Fredrik Edfors[3], Linn Fagerberg[3], Mathias Uhlén[3], Göran Bergström[4,5], Leah J. Martin[6], Jonathan W. Martin[1,2]*

[1]Department of Environmental Science, Stockholm University, Stockholm 106 91, Sweden

[2]National Facility for Exposomics, Metabolomics Platform, Science for Life Laboratory, Stockholm University, Solna 171 65, Sweden

[3]Department of Protein Science, Science for Life Laboratory, KTH-Royal Institute of Technology, Stockholm 100 44, Sweden

[4] Department of Molecular and Clinical Medicine, Institute of Medicine, Sahlgrenska Academy, University of Gothenburg, Gothenburg 40530, Sweden

[5] Department of Clinical Physiology, Sahlgrenska University Hospital, Region Västra Götaland, Gothenburg 413 45, Sweden

[6]Independent researcher. Stockholm 112 33, Sweden

Corresponding author: **Jonathan W. Martin**; Email: jon.martin@aces.su.se

This file contains:

Supplementary Methods p. S2-S4

Supplementary Figures p. S5-S34

Supplementary Table S5 p. S35-S36

Supplementary Tables S1-S4 and S6-S8

(separate file; XSLX)

Supplementary Methods

Sample Preparation. Plasma samples were prepared and analyzed following a combined targeted and untargeted chemical exposomics method described by Sdougkou et al.[1] Briefly, 200 µL plasma aliquots were placed in 2 mL tubes and fortified with 10 µL of isotopically-labelled internal standard mixture (34 labelled environmental contaminants) in methanol (MeOH; Optima LC/MS Grade, Thermo Scientific) (final concentration 1 ng/mL of each). Protein precipitation was by adding 800 µL acetonitrile (Optima LC/MS Grade, Fisher Chemical) containing 0.5% citric acid (CA) (BioUltra, anhydrous, ≥ 99.5%), vortexing for 20 s at 4 °C for 20 min, and centrifugation (20,800 x g) at 4 °C for 10 min. Supernatants were loaded to HybridSPE-Phospholipid cartridges (500 mg/6 mL, Merck) prewashed with 12 mL MeOH and 12 mL ACN containing 0.5% CA. Elution was with 1 mL ACN containing 0.5% CA, followed by 2 mL MeOH containing 1% ammonium formate (LiChropur, ≥ 99.0%) into 15 mL tubes. The pH of the extracts was adjusted from approximately 3 to 6.5 by adding 40 µL of 25% ammonia solution (LiChropur, LC-MS grade), and centrifuging for 10 min at 4,300 x g at room temperature. Supernatants were transferred to 5 mL tubes, evaporated to 100 µL under nitrogen flow and ultrasonicated for 5 min. A final rinse of the tubes with 100 µL MeOH was performed to reach a final extract volume of 200 µL, followed by centrifugal filtration (10,600 x g) for 10 min (0.2 µm nylon filters, Thermo Scientific). The final extracts were transferred to amber glass vials and spiked with 10 µL of diuron-d6 solution (final concentration 4 ng/mL) to correct for extract volume variations and to monitor instrumental performance.

LC-HRMS Analysis. Measurements were conducted by ultrahigh pressure LC (Ultimate 3000, Thermo Scientific) with HRMS acquisition (Q Exactive Orbitrap HF-X, Thermo Scientific) in positive and negative electrospray ionization mode (ESI+ and ESI-) as previously described.[1] Spectral acquisition alternated between full scan (i.e. MS1; 90-1000 mass-to-charge ratio (m/z), 120,000 nominal resolution) and data-independent MS/MS acquisition (DIA) (i.e. MS2; 30,000 nominal resolution, product ion scan range starting from 50 Da) with four m/z precursor windows of equal size (237 Da), with each window overlapping by 10 Da. Data-dependent acquisition (DDA) with an inclusion list of precursor ions was used for analyte confirmations. Injection volumes were 20 µL, corresponding to 20 µL plasma-equivalents on-column, and chromatography was at 40 °C on an Acquity BEH C18 column (130 Å, 1.7 µm, 3 x 100 mm, Waters) with an Acquity BEH C18 1.7 µM vanguard pre-column. Upstream of the injector, an Acquity BEH C18 column (130 Å, 1.7 µm, 3 x 30 mm, Waters) was placed to separate instrumental background from sample analytes. A binary gradient elution at 0.4 mL/min used mobile phases (A) water (Optima LC/MS Grade, Thermo Scientific) containing 1 mM ammonium fluoride[2] (Honeywell Fluka, ≥ 98.0%), and (B) 100% MeOH. The elution gradient started at 5% B, linearly

increased to 100% B by 15 min, held until 22 min, returning to initial conditions with 4 min equilibration.

Analyte Quantification. Peak areas were integrated using Xcalibur Quan Browser (Thermo Scientific, v.4.1), and solvent-based calibration curves with internal standards (9 points, 0.01-100 ng/mL) were used to quantify the targeted analytes. For three targeted PFAS analytes (perfluorooctane sulfonate (PFOS), perfluorohexane sulfonate (PFHxS), and perfluoroheptane sulfonate (PFHpS)) when branched isomers were detected in plasma these were quantified separately from the corresponding linear isomers. In each batch the calibration curves were run 3 times (beginning, middle, end). Pooled Swedish reference plasma was also run multiple times in each injection sequence to support reference standardization[3] quantification of steroid hormones, and to enable retrospective semi-quantification of discovered substances in the untargeted analysis. The Swedish pooled plasma was produced by pooling heparin plasma samples from 953 Swedish individuals (male and female) from the Västerbotten Intervention Programme (VIP) based in the Northern Sweden Biobank. To support reference standardization, standard addition curves were employed to quantify the discovered substances in the pooled Swedish reference sample. For data summaries and statistics, when analytes were detected at concentrations lower than the respective MLOQ, the analyte concentrations were substituted by MLOQ/2, and when analytes were non-detect, the concentrations were substituted by MLOQ/4.

Untargeted Data Filtering and Reduction. In MS-DIAL only features with peak areas 5 times higher than the corresponding procedural blanks (2 blanks per batch) were kept (i.e. sample maximum / blank average > 5). In further processing and after all data normalization, the ratio of each sample's peak area to the average procedural blank peak area was computed for each feature; 0.1 was added to the average procedural blank area of each feature to avoid 0 in the denominator when there was no blank response. For each feature, only samples with peak areas at least 5 times higher than the corresponding blank average were considered; areas in each sample that did not pass this threshold were set to 0. Average areas in blank (when detectable) were subtracted from peak areas in samples which passed the threshold. This additional blank filtering / subtraction step constrained potential artefacts in the final dataset that would lead to a misleading higher detection frequency (DF). As a final data reduction step, highly correlating annotated features (Pearson correlation coefficient (r) > 0.95, p-value < 0.001) with RTs differing by a maximum of 0.1 min, and with identical peak shapes (assessed by visual inspection) were considered as originating from the same analyte (e.g. produced by in-source fragmentation, or an adduct); the feature with lowest average peak area was discarded.

Quality Control Notes. All samples were prepared in a positive pressure clean laboratory with high efficiency particulate filtration. The 276 plasma samples were extracted and analyzed in eight batches.

Samples from the same individuals were analyzed within the same batch, but were randomized in the injection sequence. Procedural blanks were prepared (2 blanks per batch) and treated in the same manner as the experimental plasma samples. Targeted analytes were in general absent in procedural blanks, however peaks were detectable for the plasticizer chemicals and metabolites (bisphenol A, monoethyl phthalate, monoisobutyl phthalate), personal care product chemicals (methylparaben, propylparaben, and oxybenzone), flame retardants or metabolites of them (diphenyl phosphate, tris-2-butoxyethyl phosphate) and the insecticide DEET. For quantification of the above targeted analytes only samples with a peak area of at least 4 times higher than the blank signal were considered. The blank concentration was then subtracted from the sample concentration. Instrumental blanks consisting of clean solvent were also run multiple times in the injection sequence to monitor and prevent carryover.

A targeted analyte that was detected with high detection frequency (55%) but excluded from further data processing and visualizations was bisphenol A (BPA) (median concentration in samples 2 ng/mL, max 401.5 ng/mL). BPA in humans and animals is rapidly metabolized to a glucuronide of BPA, thus only low levels are expected in blood of healthy individuals.[4,5] Since no field blank was available (representing sampling and storage of samples prior being received), it was considered appropriate to discard BPA quantifications, despite being well above the blank contamination level. An additional proof for discarding this analyte was its strong correlation with the Level 2 annotated feature bisphenol A- (2,3-dihydroxypropyl)-glycidyl ether (BADGE-H2O) (Pearson coefficient 0.92, p-value < 0.001, **Figure S29**). BADGE-H2O is a hydrolysis product of bisphenol A diglycidyl ether (BADGE), which is often used as a monomer in the production of epoxy-based polymers, as well as an additive for the elimination of surplus hydrochloric acid in polyvinyl chloride (PVC) organosol production.[6] BADGE can transform to BADGE-H2O in the environment,[6] and similar to BADGE, BADGE-H2O is unstable in biological matrices.[7]

Intraclass Correlation Coefficient Calculation. To calculate the intraclass correlation coefficient (ICC) for molecular profiles reported by Tebani et al.,[8] only individuals present in the exposomics study reported here (n=46) were included. Since, not all 46 individuals and /or all visits were included in each molecular profile in Tebani et al.,[8] deviating numbers of samples were included here for the ICC calculation for each molecular profile. More specifically, 22 individuals (4 visits per individual) were used for the lipidome, 46 individuals (4 visits per individual) were used for the metabolome, 44 individuals (6 visits per individual) were used for the proteome and 44 individuals (4 visits per individual) were used for the microbiome ICC calculation.

Supplementary Figures

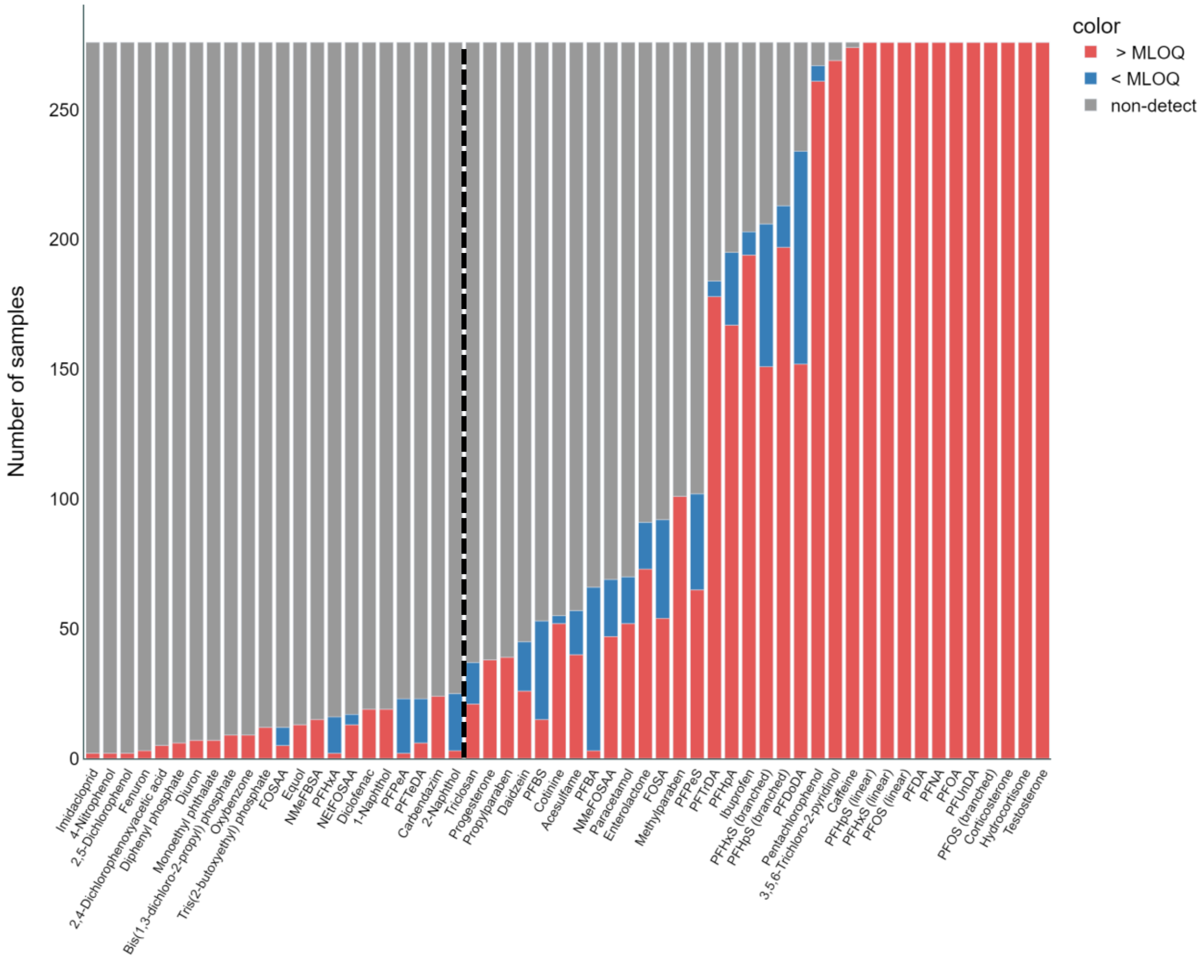

Figure S1: Detected target analytes ordered by detection frequency from left to right (1-100%, respectively) among all 276 S3WP plasma samples. Detections above the MLOQ are shown in red, detections below MLOQ are shown in blue, and non-detects are shown in gray. All samples above detection limits (blue and red) were counted towards the detection frequency. The dotted line separates analytes with detection frequency > 10%. For statistical analysis, detections below MLOQ (blue) were substituted by MLOQ/2, and non-detects (gray) were substituted by MLOQ/4.

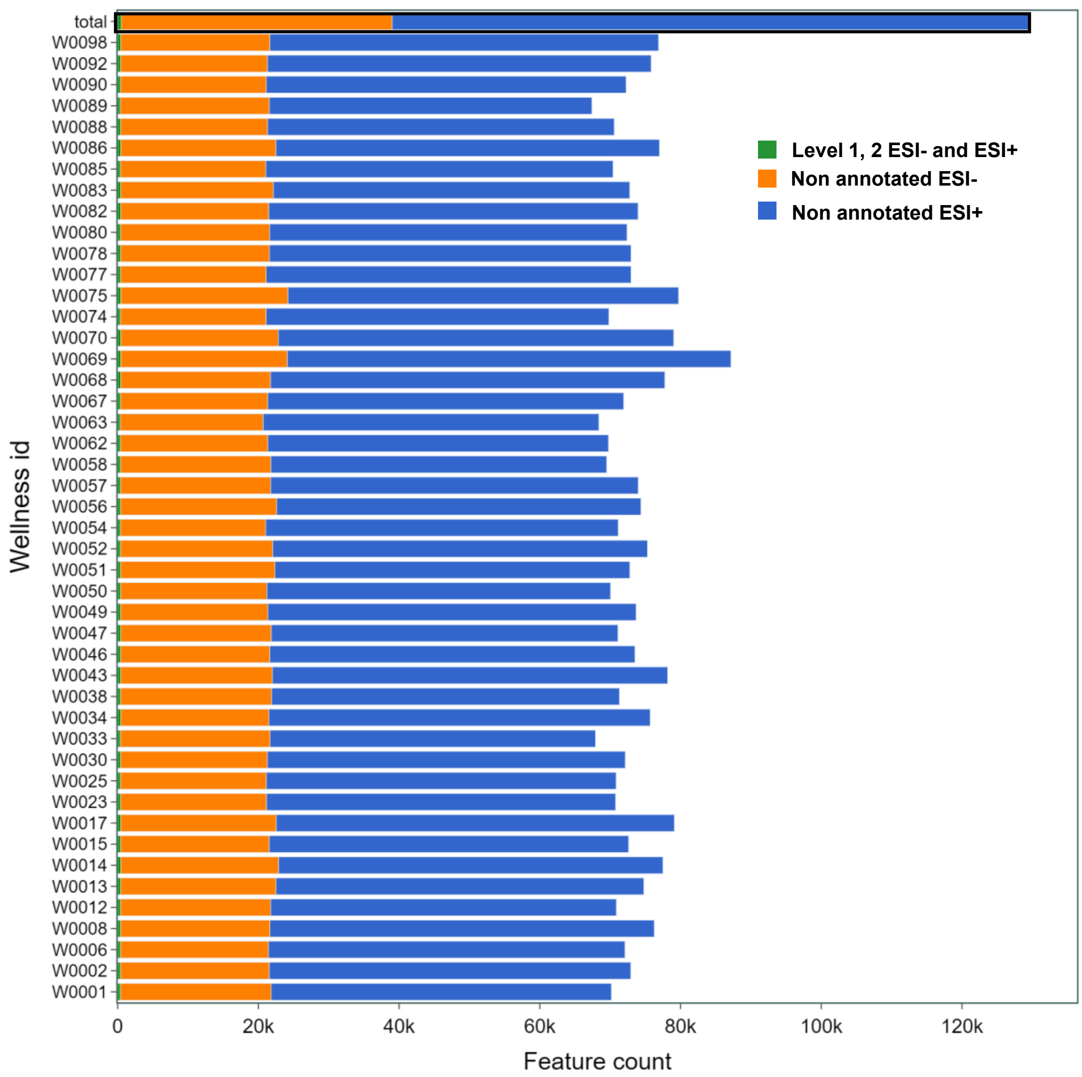

Figure S2: Number of annotated features (green, ESI- and ESI+ Level 1 and Level 2) and non-annotated features detected in ESI- (orange) and ESI+ (blue) by untargeted analysis. The feature counts in each category for each individual are a cumulative total number of features detected among all visits, and the top bar ("total") indicates the cumulative total number of features detected among all individuals and all visits.

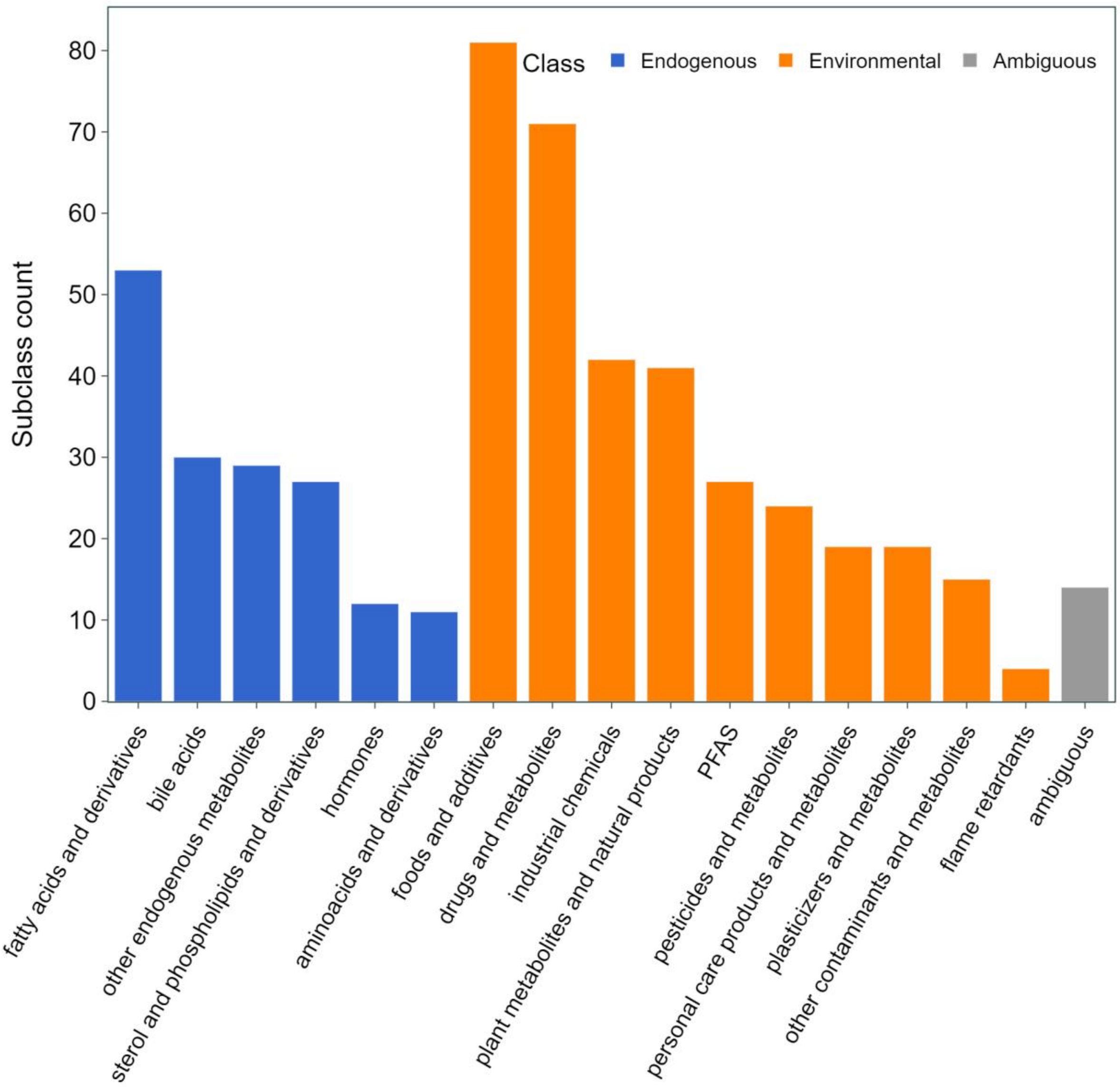

Figure S3: Total number of targeted and annotated untargeted analytes by chemical class (blue = endogenous, orange = environmental, gray = ambiguous) and various subclasses (x-axis labels)

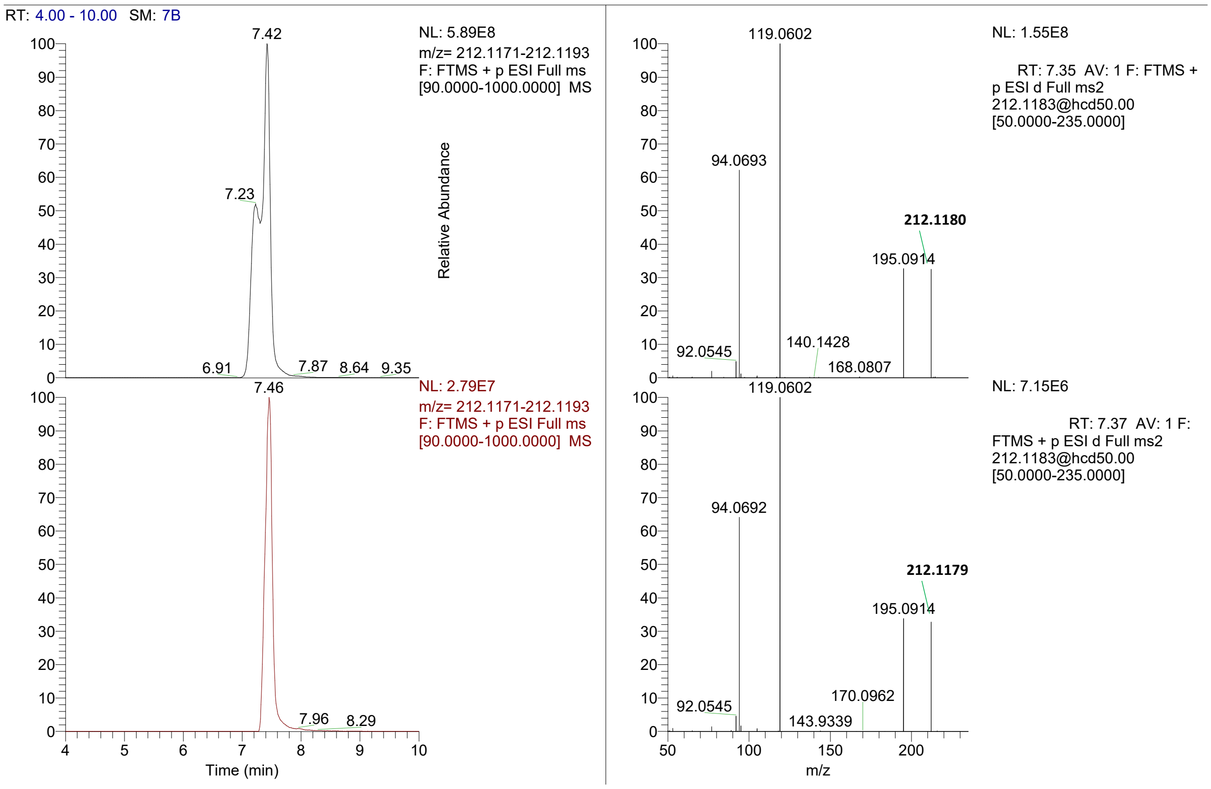

Figure S4. Extracted ion chromatogram (left) and data-dependent acquisition (DDA) spectrum (right) for 1,3-diphenylguanidine in individual plasma (top) and standard solution (bottom).

144 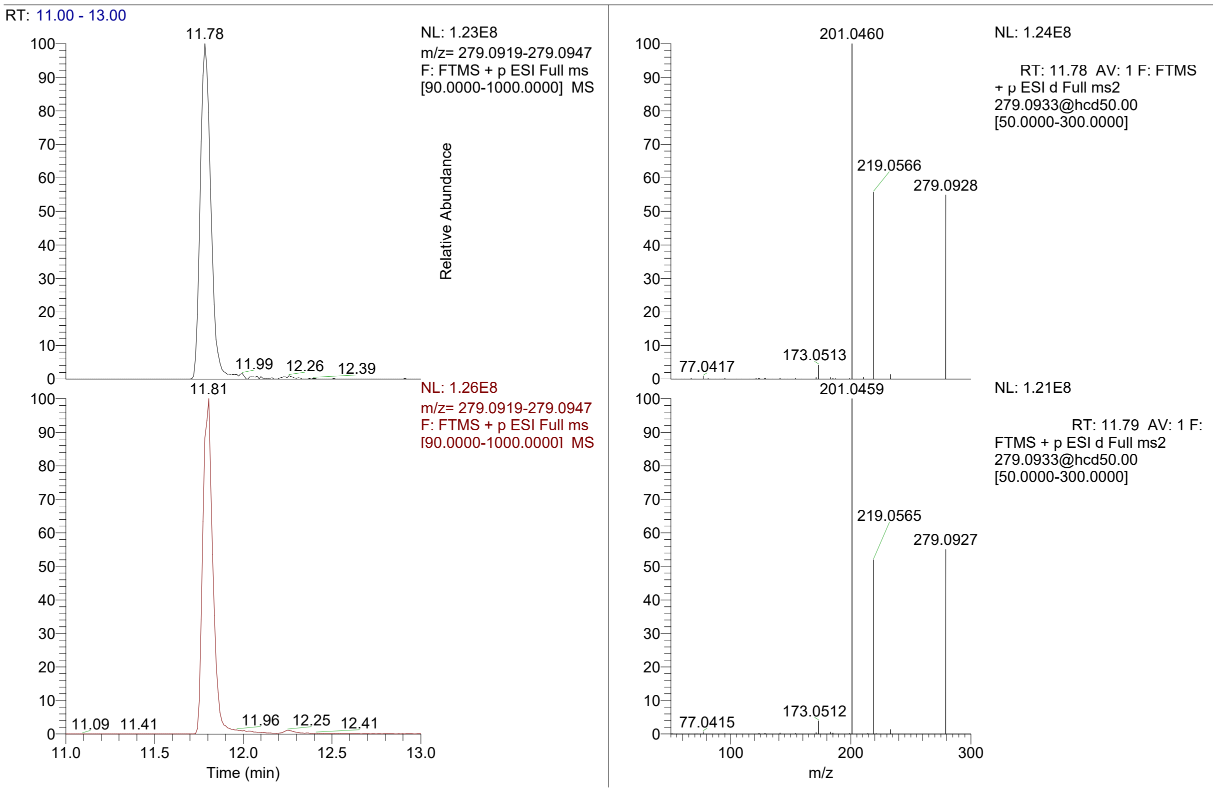

145 **Figure S5.** Extracted ion chromatogram (left) and data-dependent acquisition (DDA) spectrum (right)
146 for triphenyl phosphine oxide in individual plasma (top) and standard solution (bottom).

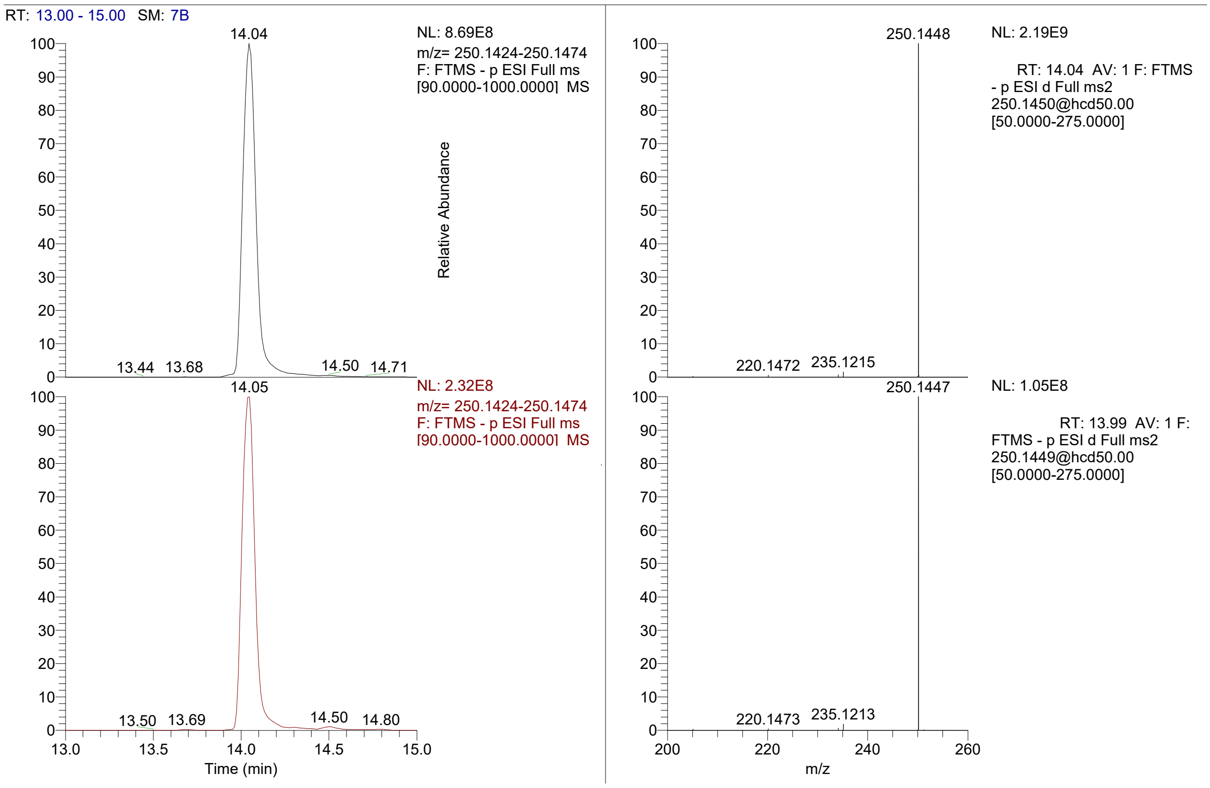

Figure S6. Extracted ion chromatogram (left) and data-dependent acquisition (DDA) spectrum (right) for 2,6-di-tert-butyl-4-nitrophenol in individual plasma (top) and spiked pooled plasma (bottom).

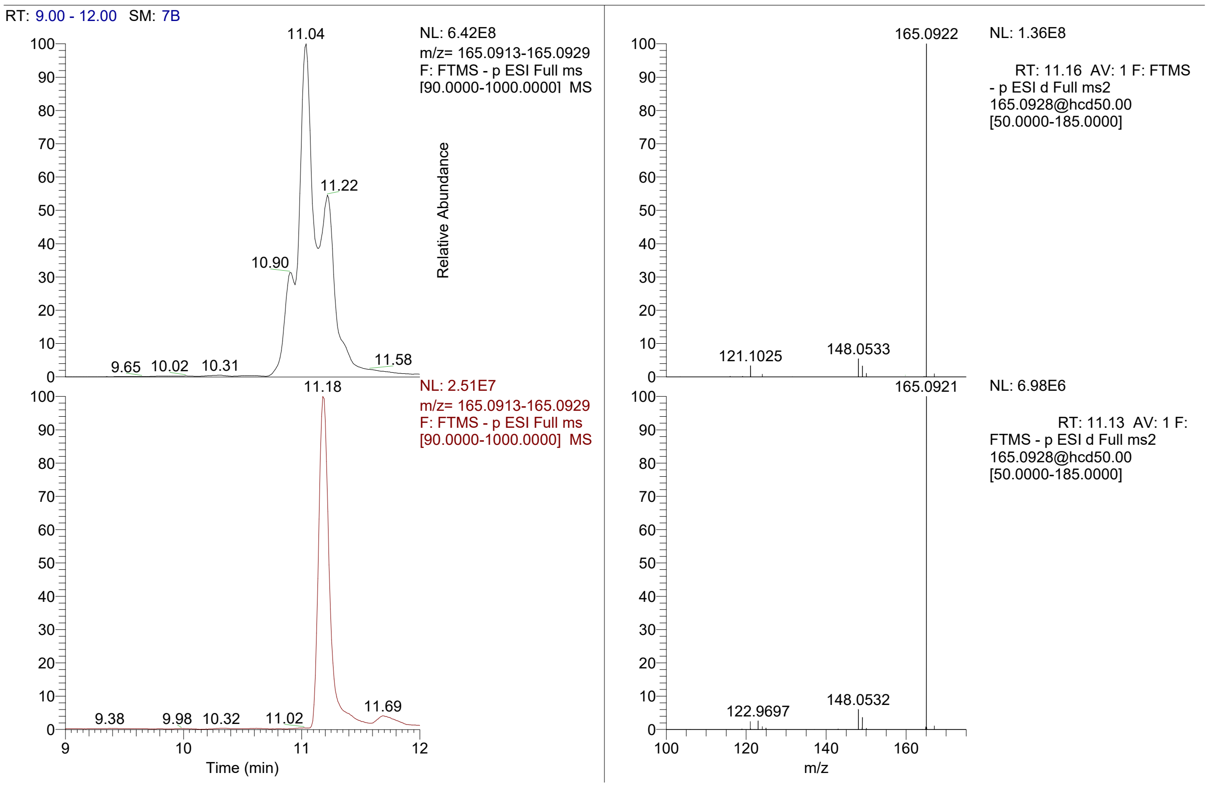

Figure S7. Extracted ion chromatogram (left) and data-dependent acquisition (DDA) spectrum (right) for 4-tert-butylpyrocatechol in individual plasma (top) and standard solution (bottom).

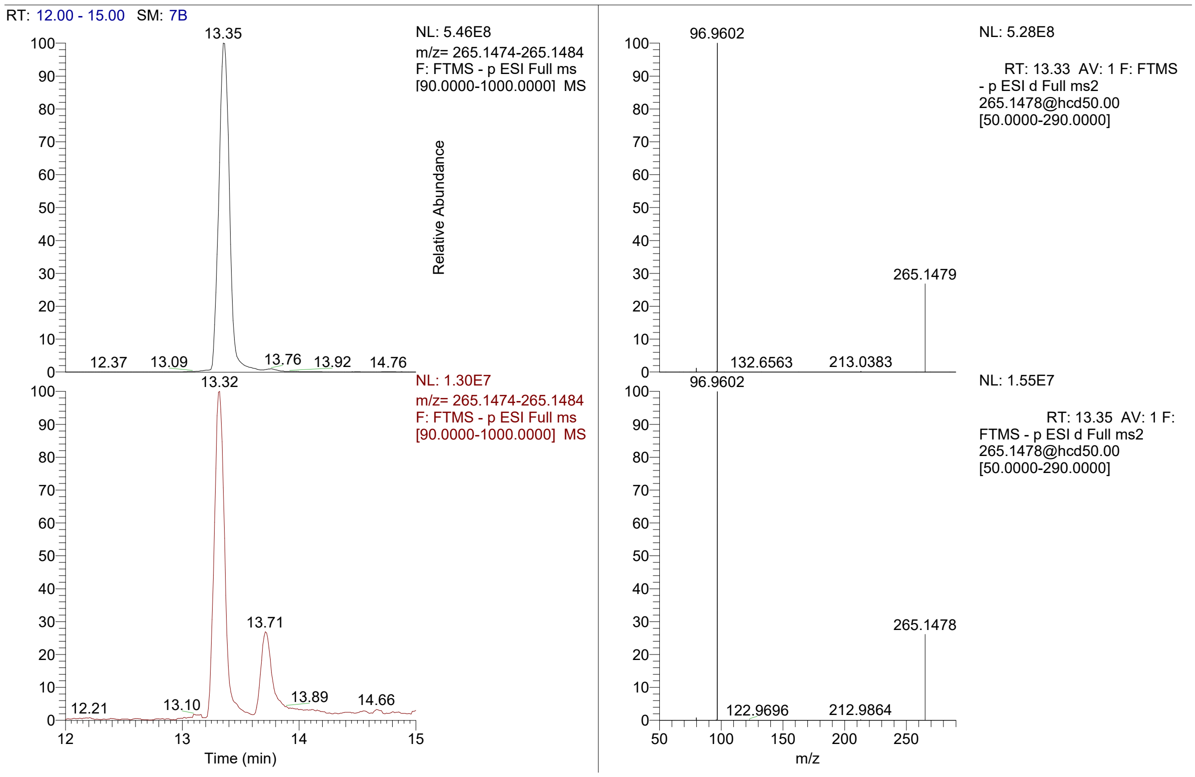

Figure S8. Extracted ion chromatogram (left) and data-dependent acquisition (DDA) spectrum (right) for sodium lauryl sulphate in individual plasma (top) and standard solution (bottom). A background peak elutes at 13.71 in the standard solution and 13.76 in individual plasma.

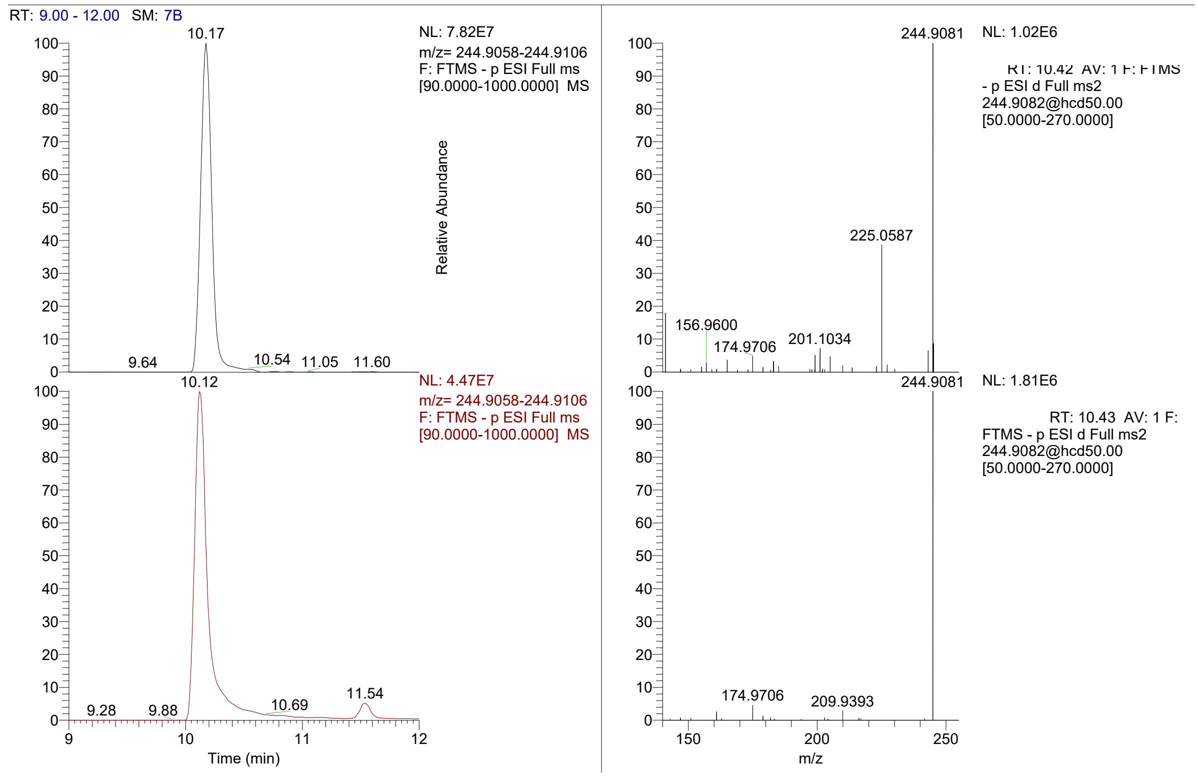

Figure S9. Extracted ion chromatogram (left) and data-dependent acquisition (DDA) spectrum (right) for chlorothalonil-4-hydroxy in individual plasma (top) and standard solution (bottom).

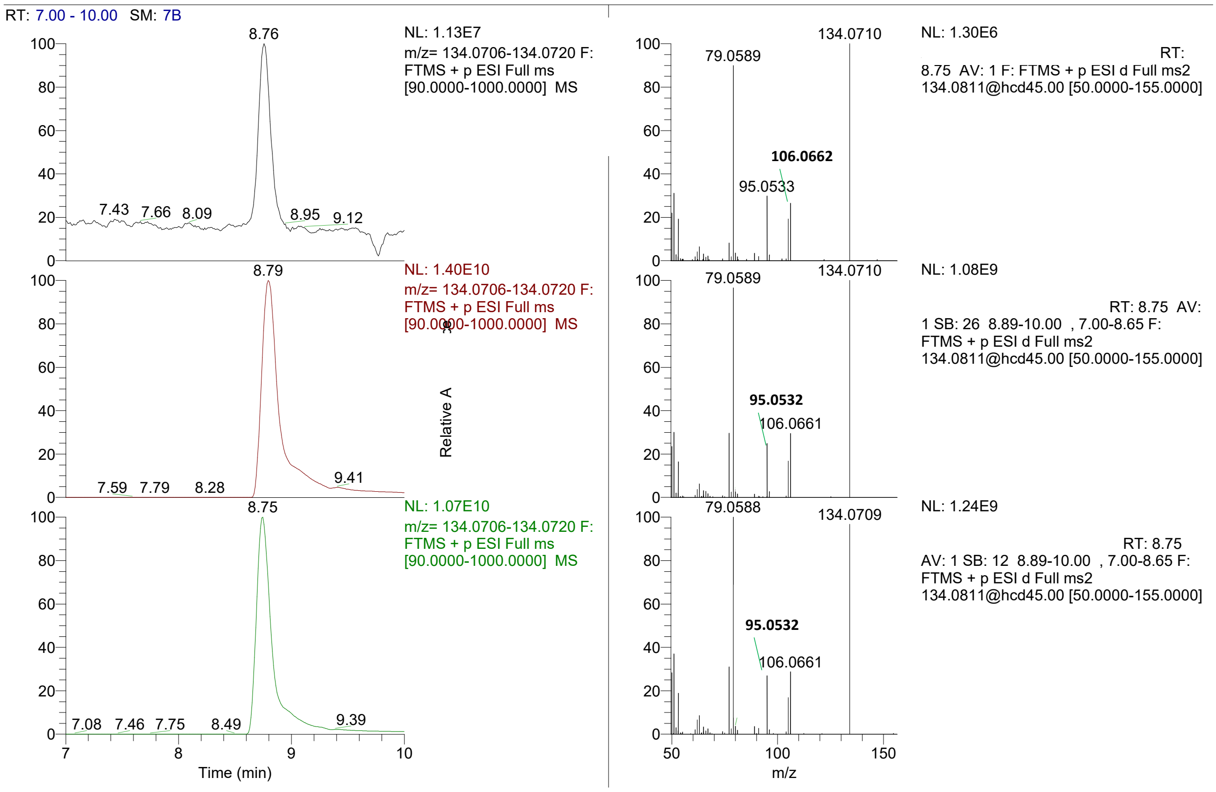

Figure S10. Extracted ion chromatogram (left) and data-dependent acquisition (DDA) spectrum (right) for the sum of 4- and 5-methyl-1H-benzotriazole in individual plasma (top) and standard solution (middle: 4-methyl-1H-benzotriazole, bottom: 5-methyl-1H-benzotriazole).

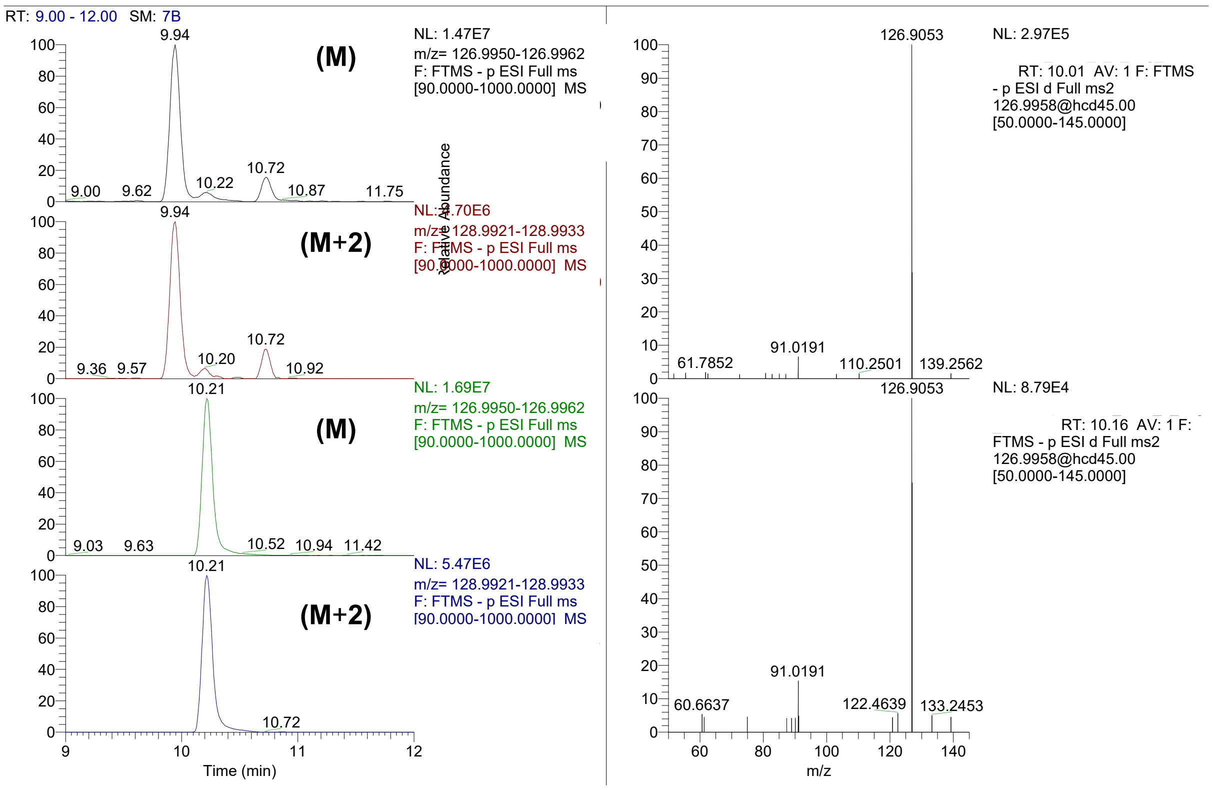

Figure S11. Extracted ion chromatogram (EIC) (left) and data-dependent acquisition (DDA) spectrum (right) for 4-chlorophenol in individual plasma (top two EICs and top spectrum) and standard solution (bottom two EICs and bottom spectrum). The EICs are shown in both sample and standard for the M and M+2 ion, corresponding to the chlorine isotopes with atomic masses of 35 Da and 37 Da. 4-chlorophenol elutes at 10.20 min, while chlorinated isomers elute at 9.94 and 10.72 min in the individual plasma.

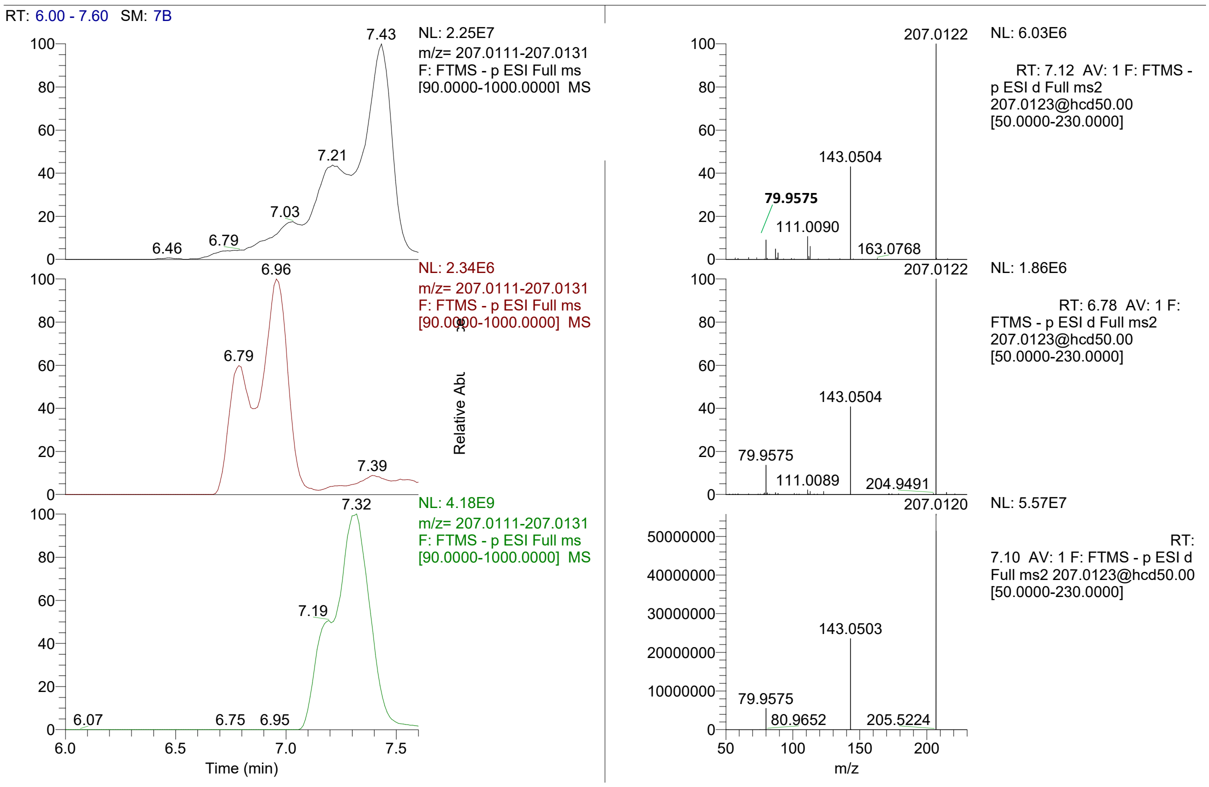

Figure S12. Extracted ion chromatogram (left) and data-dependent acquisition (DDA) spectrum (right) for the sum of 1- and 2- naphthalenesulfonate in individual plasma (top) and standard solution (middle: 1- naphthalenesulfonate, bottom: 2- naphthalenesulfonate).

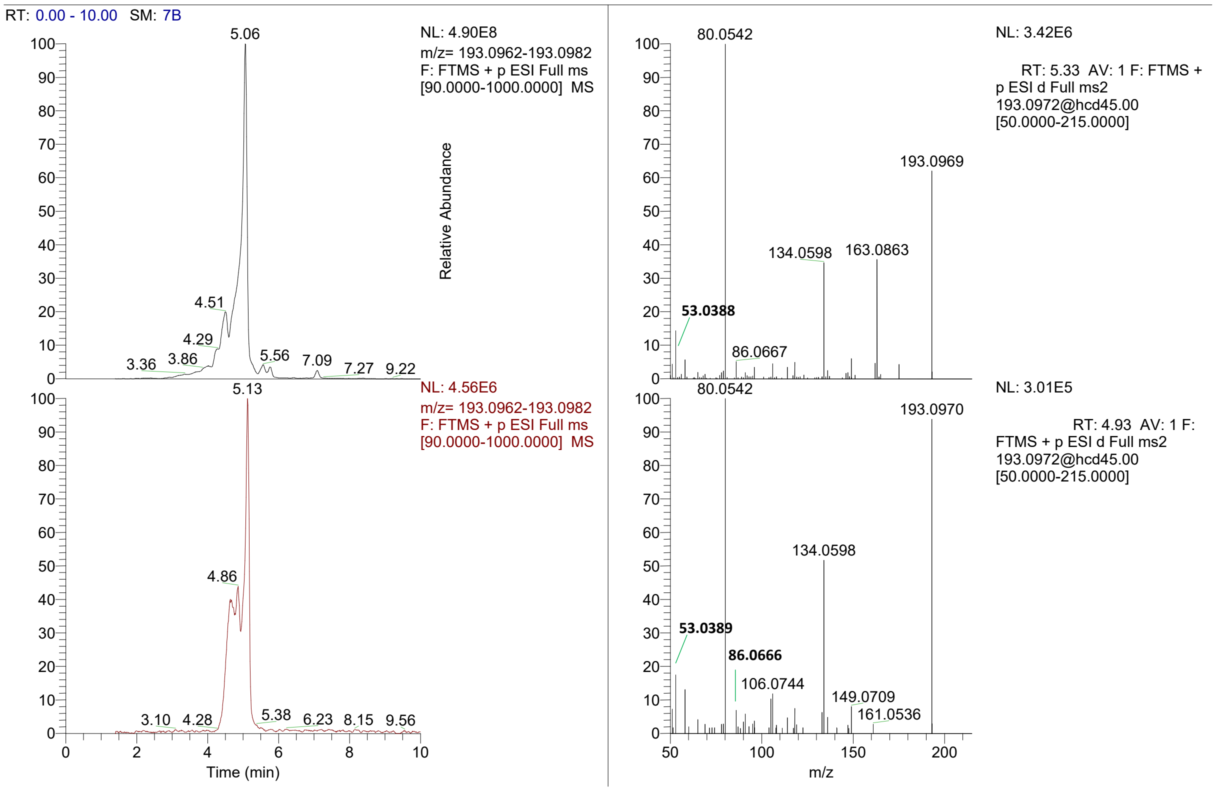

Figure S13. Extracted ion chromatogram (left) and data-dependent acquisition (DDA) spectrum (right) for 3-hydroxycotinine in individual plasma (top) and standard solution (bottom).

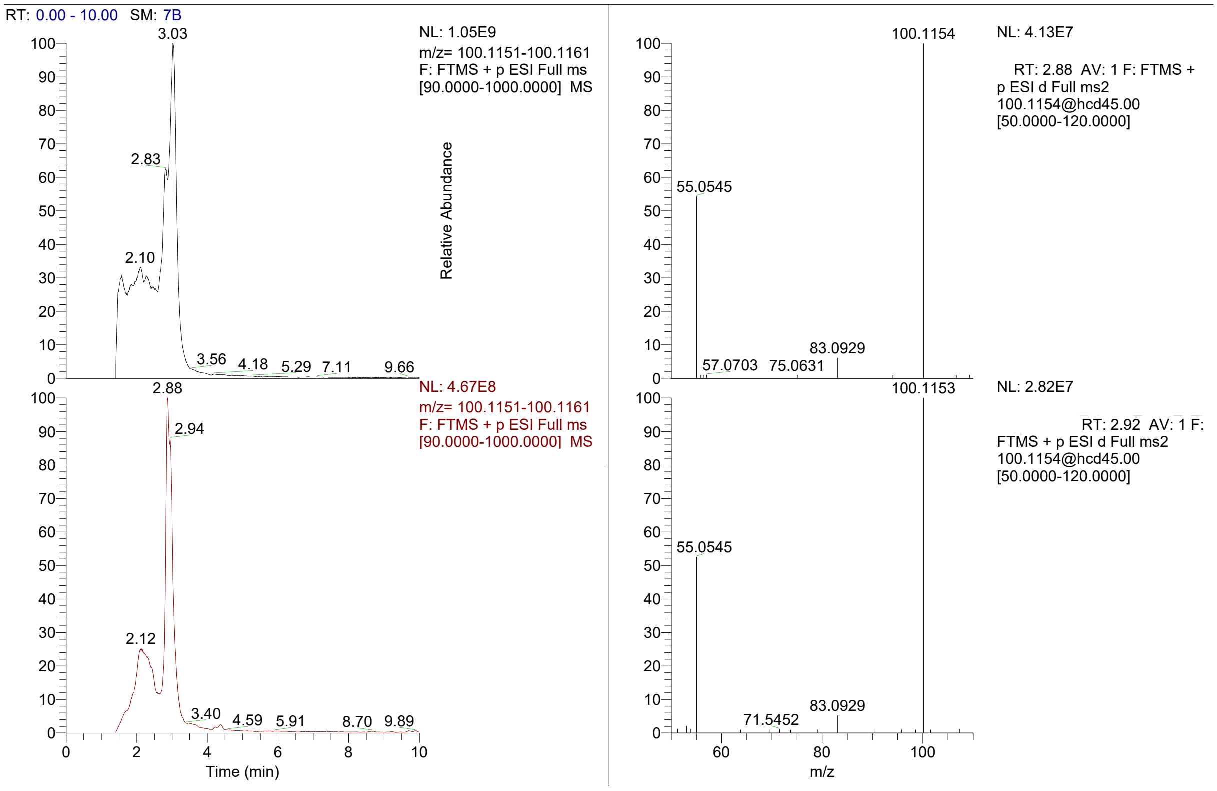

178

179 **Figure S14.** Extracted ion chromatogram (left) and data-dependent acquisition (DDA) spectrum (right)
180 for cyclohexylamine in individual plasma (top) and standard solution (bottom).

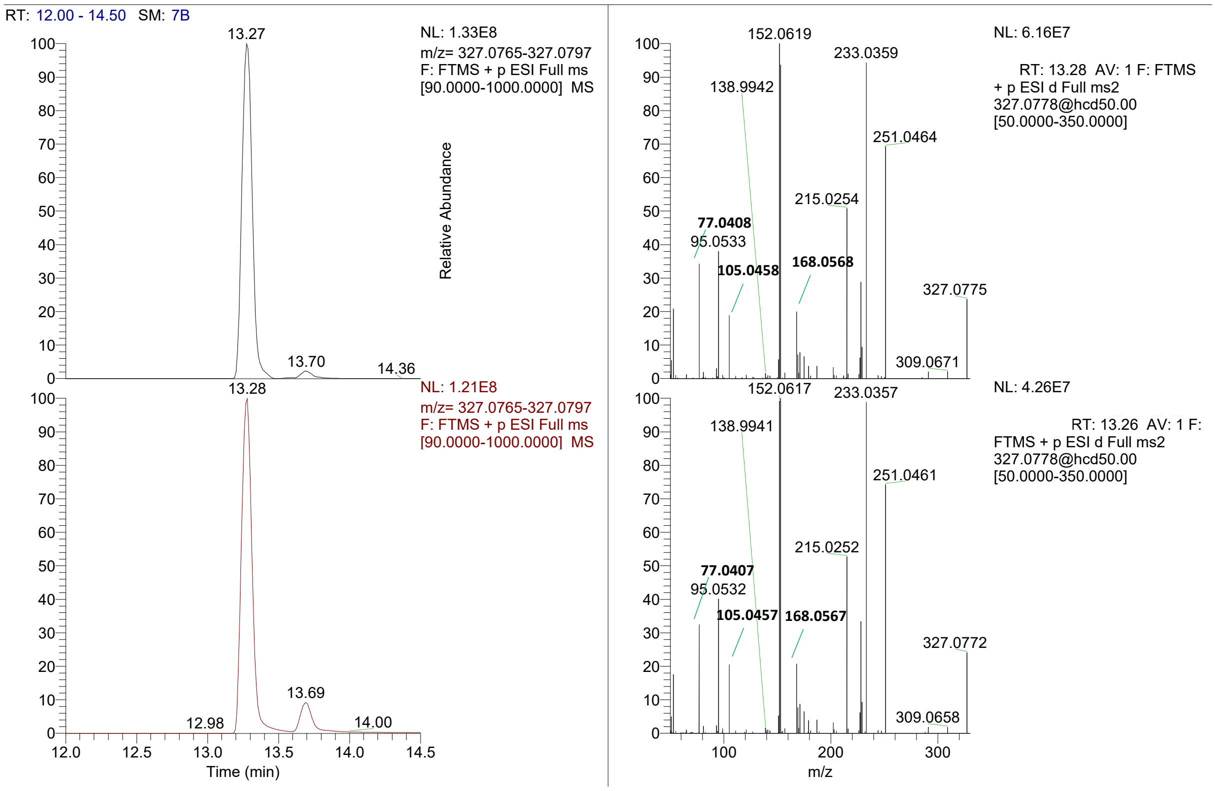

Figure S15. Extracted ion chromatogram (left) and data-dependent acquisition (DDA) spectrum (right) for triphenyl phosphate in individual plasma (top) and standard solution (bottom).

184 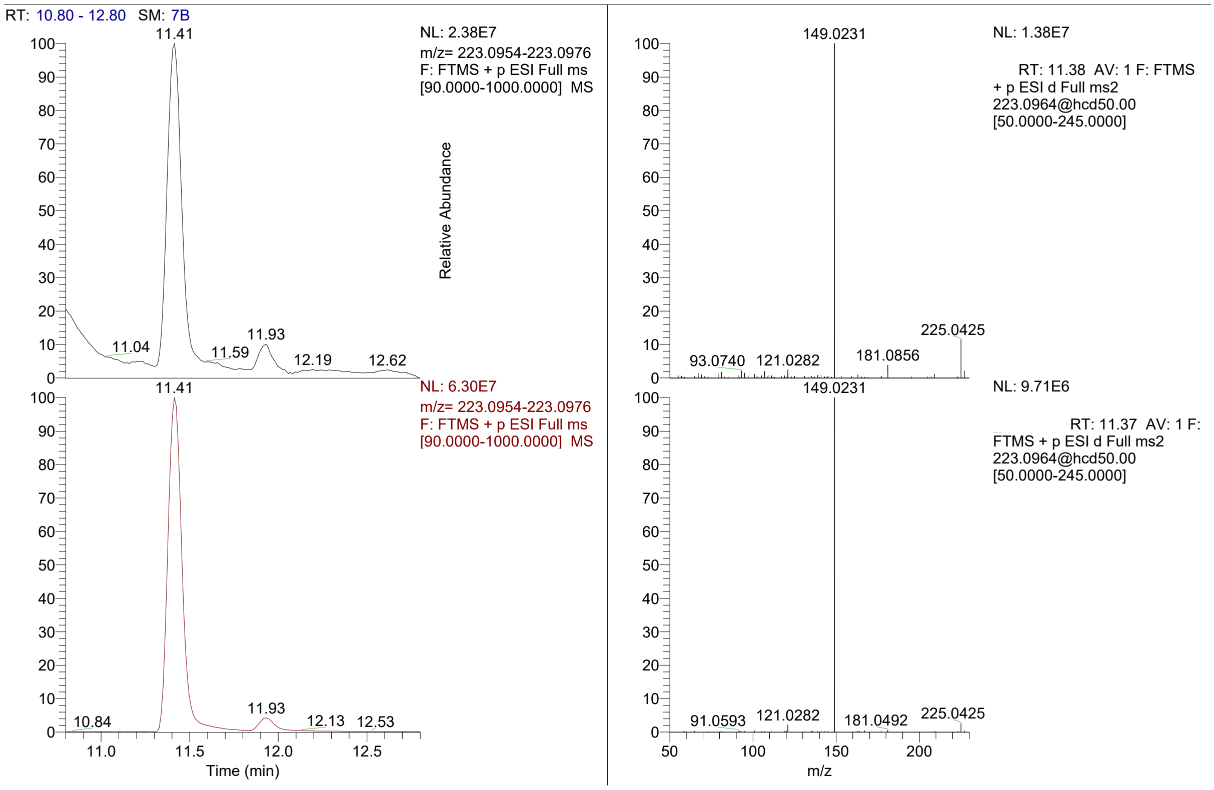

185 **Figure S16.** Extracted ion chromatogram (left) and data-dependent acquisition (DDA) spectrum (right)
186 for diethyl phthalate in individual plasma (top) and standard solution (bottom).

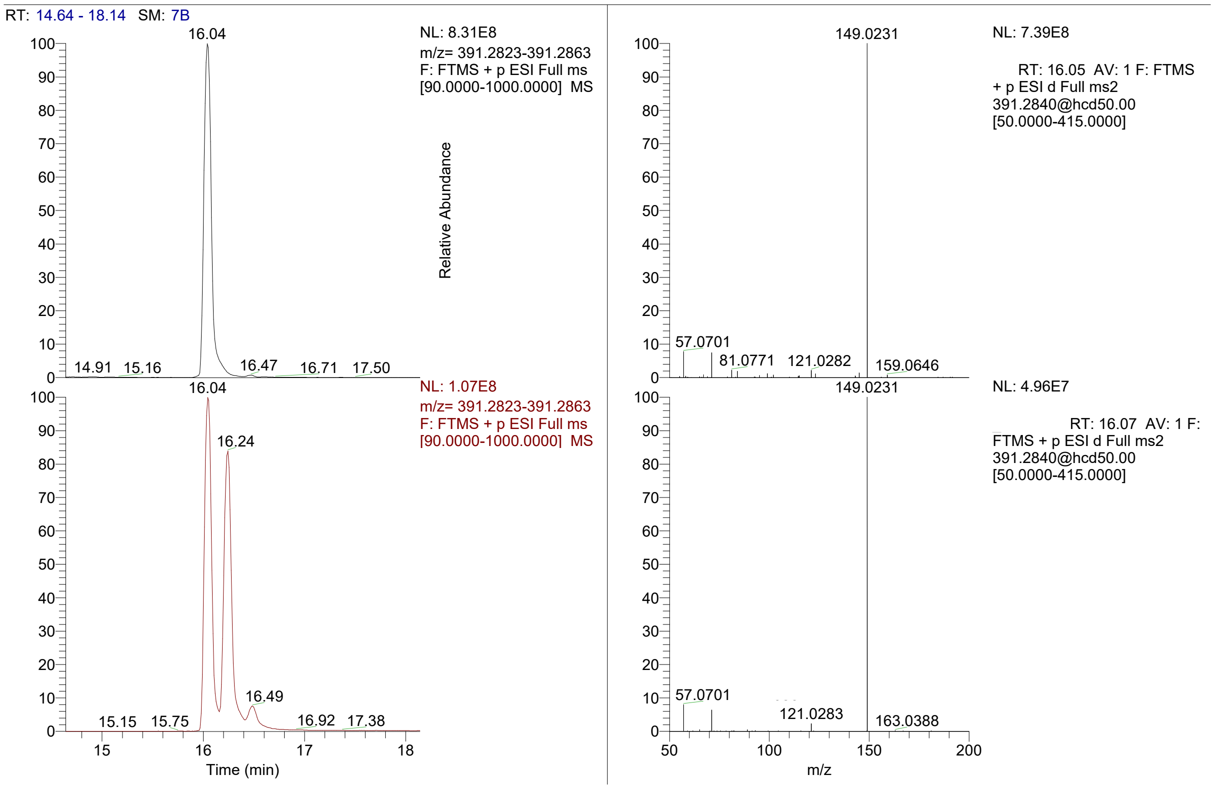

Figure S17. Extracted ion chromatogram (left) and data-dependent acquisition (DDA) spectrum (right) for Bis(2-ethylhexyl) phthalate (DEHP) in individual plasma (top) and standard solution (bottom). In the standard solution di-n-octyl phthalate (also spiked) elutes at 16.24 min. A background peak elutes at 16.49 in the standard solution and 16.47 in individual plasma.

192 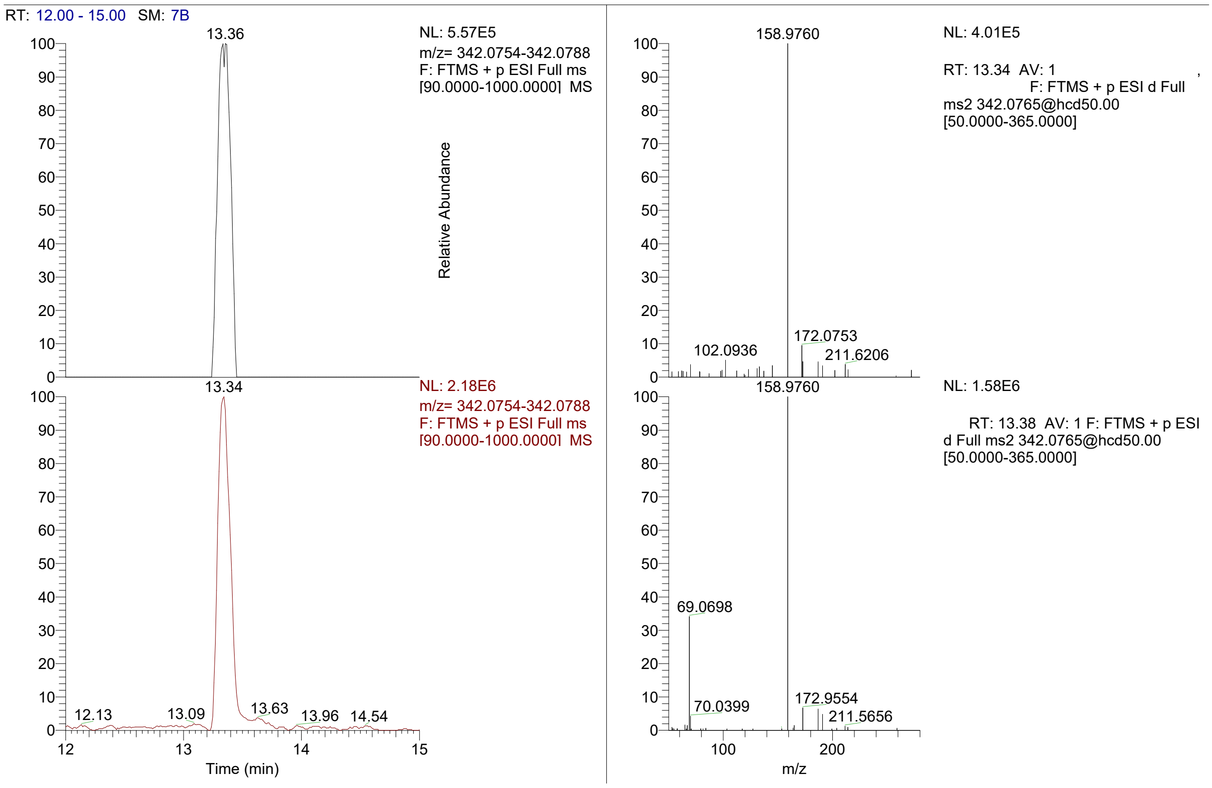

193 **Figure S18.** Extracted ion chromatogram (left) and data-dependent acquisition (DDA) spectrum (right)
194 for propiconazole in individual plasma (top) and standard solution (bottom).

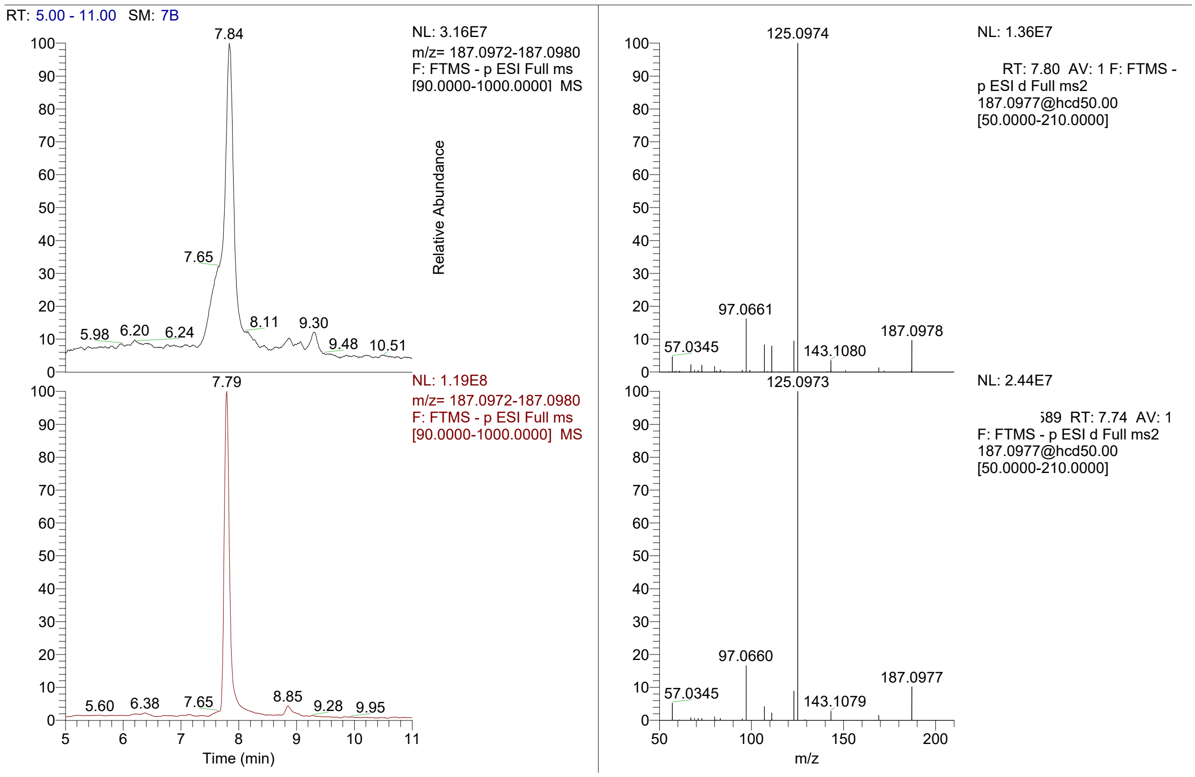

Figure S19. Extracted ion chromatogram (left) and data-dependent acquisition (DDA) spectrum (right) for azelaic acid in individual plasma (top) and standard solution.

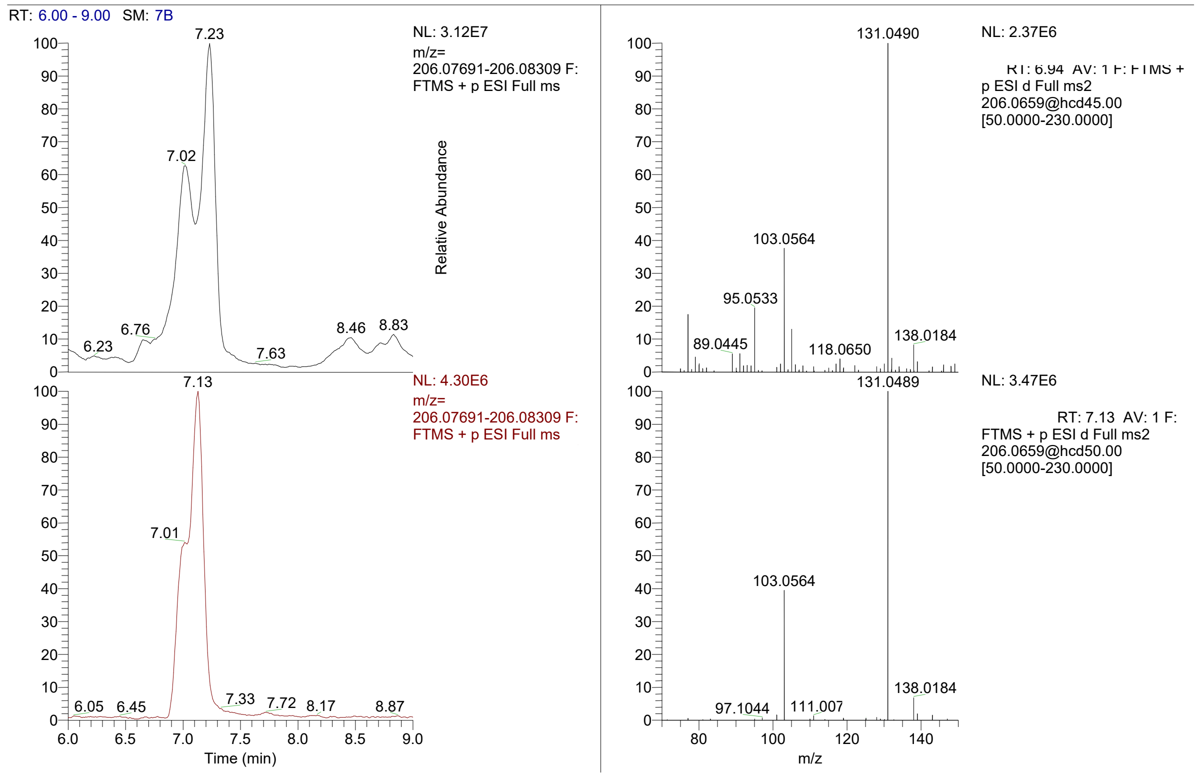

Figure S20. Extracted ion chromatogram (left) and data-dependent acquisition (DDA) spectrum (right) for N-cinnamoylglycine in individual plasma (top) and standard solution.

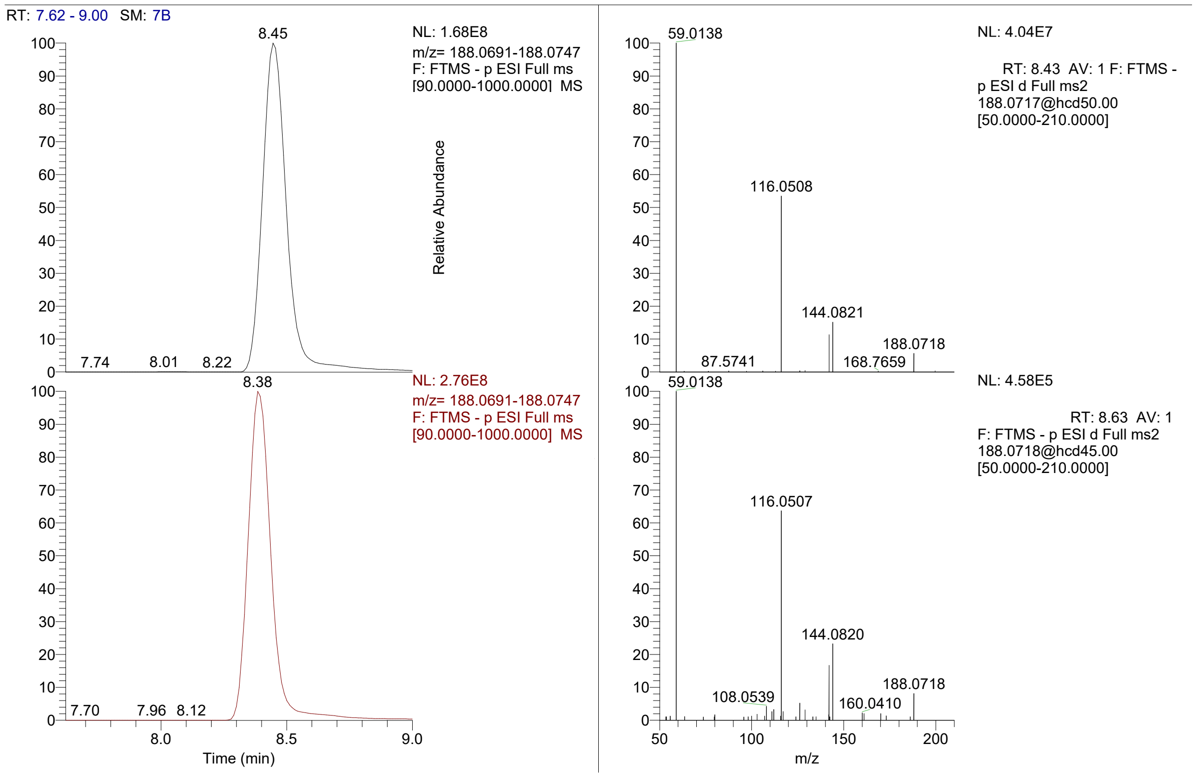

Figure S21. Extracted ion chromatogram (left) and data-dependent acquisition (DDA) spectrum (right) for indolepropionic acid in individual plasma (top) and standard solution.

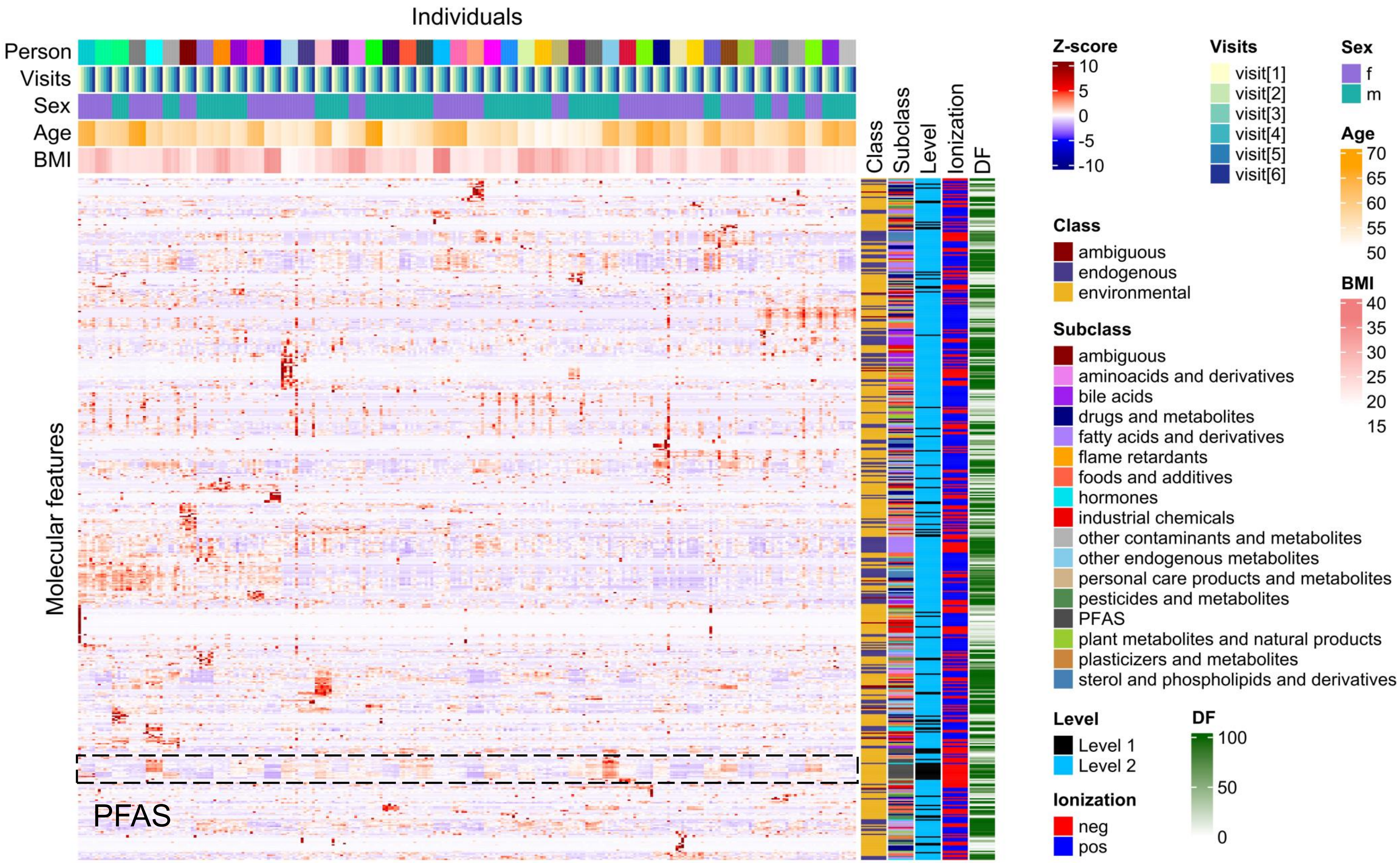
Individuals
Person
Visits
Sex
Age
BMI
Molecular features
PFAS
Class
Subclass
Level
Ionization
DF
Z-score
10
5
0
-5
-10
Visits
visit[1]
visit[2]
visit[3]
visit[4]
visit[5]
visit[6]
Sex
f
m
Age
70
65
60
55
50
BMI
40
35
30
25
20
15
Class
ambiguous
endogenous
environmental
Subclass
ambiguous
aminoacids and derivatives
bile acids
drugs and metabolites
fatty acids and derivatives
flame retardants
foods and additives
hormones
industrial chemicals
other contaminants and metabolites
other endogenous metabolites
personal care products and metabolites
pesticides and metabolites
PFAS
plant metabolites and natural products
plasticizers and metabolites
sterol and phospholipids and derivatives
Level
Level 1
Level 2
Ionization
neg
pos
DF
100
50
0

Figure S22. Hierarchical cluster analysis heatmap showing exposome profiles for 46 individuals at each of 6 visits, including 519 annotated substances (Level 1 and Level 2). The entire heatmap overview is shown where color coding of features is according to class, subclass, confidence Level of identification, ionization and detection frequency (DF). Color coding of individuals is according to visit, sex, age and BMI. The order of individuals and molecular features is according to hierarchical cluster analysis conducted for the averaged responses across visits for each individual, as shown in Figure 3. A group of clustered PFAS substances is highlighted on the heatmap.

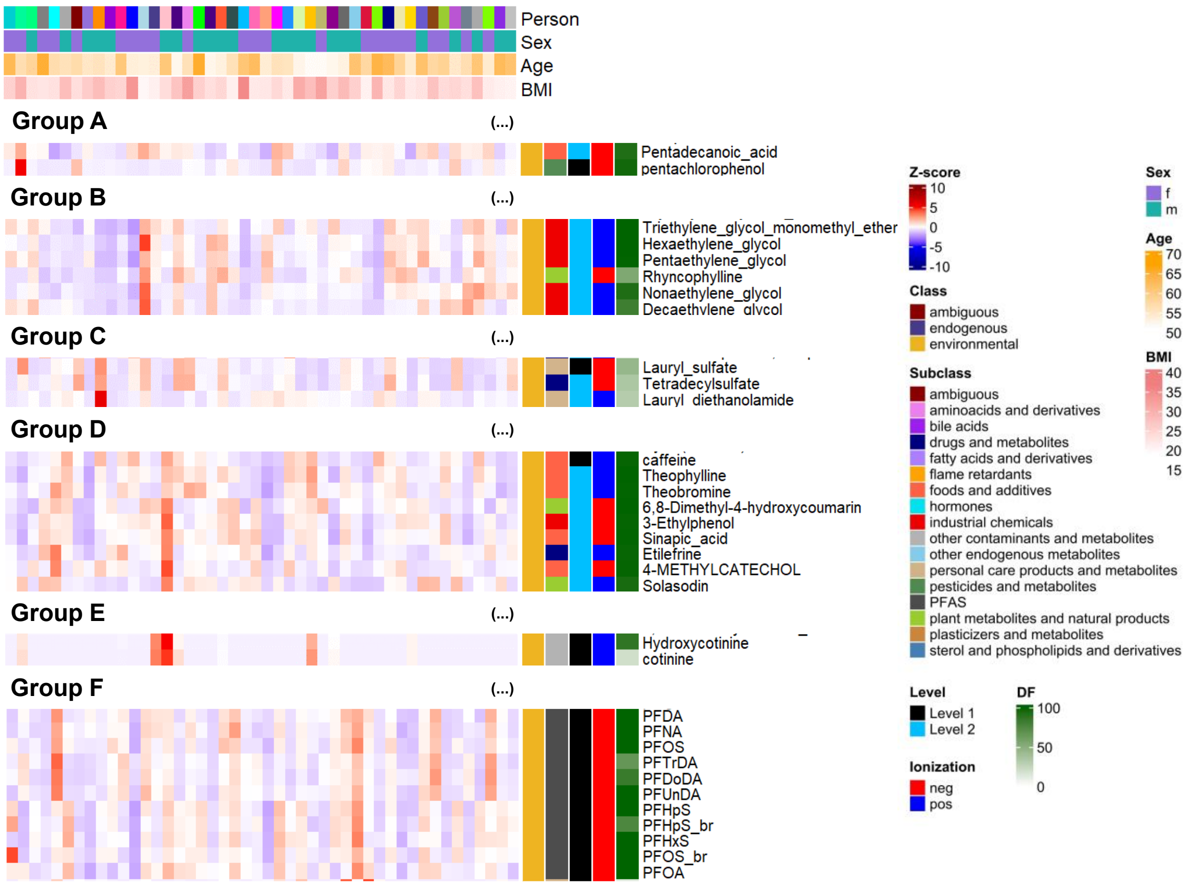

Figure S23: Zoomed-in sections of the hierarchical cluster analysis heatmap, showing the exposome profiles of 46 individuals, each averaged across the 6 clinical visits (full version in Figure 3). Groups of common co-exposures (detected in many participants) are shown.

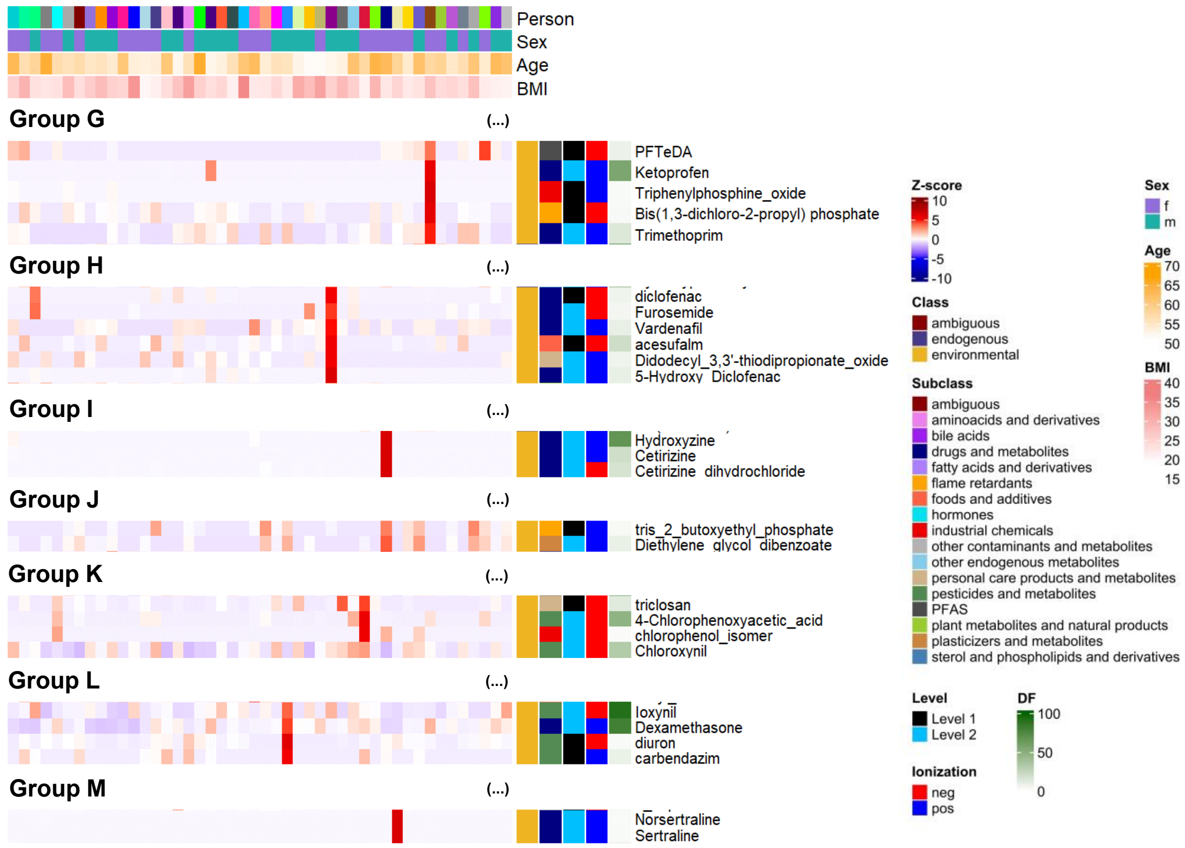

Figure S24: Zoomed-in sections of the hierarchical cluster analysis heatmap, showing the exposome profiles of 46 individuals, each averaged across the 6 clinical visits (full version in Figure 3). Groups of rare co-exposures (detected only in individuals or a small population fraction) are shown.

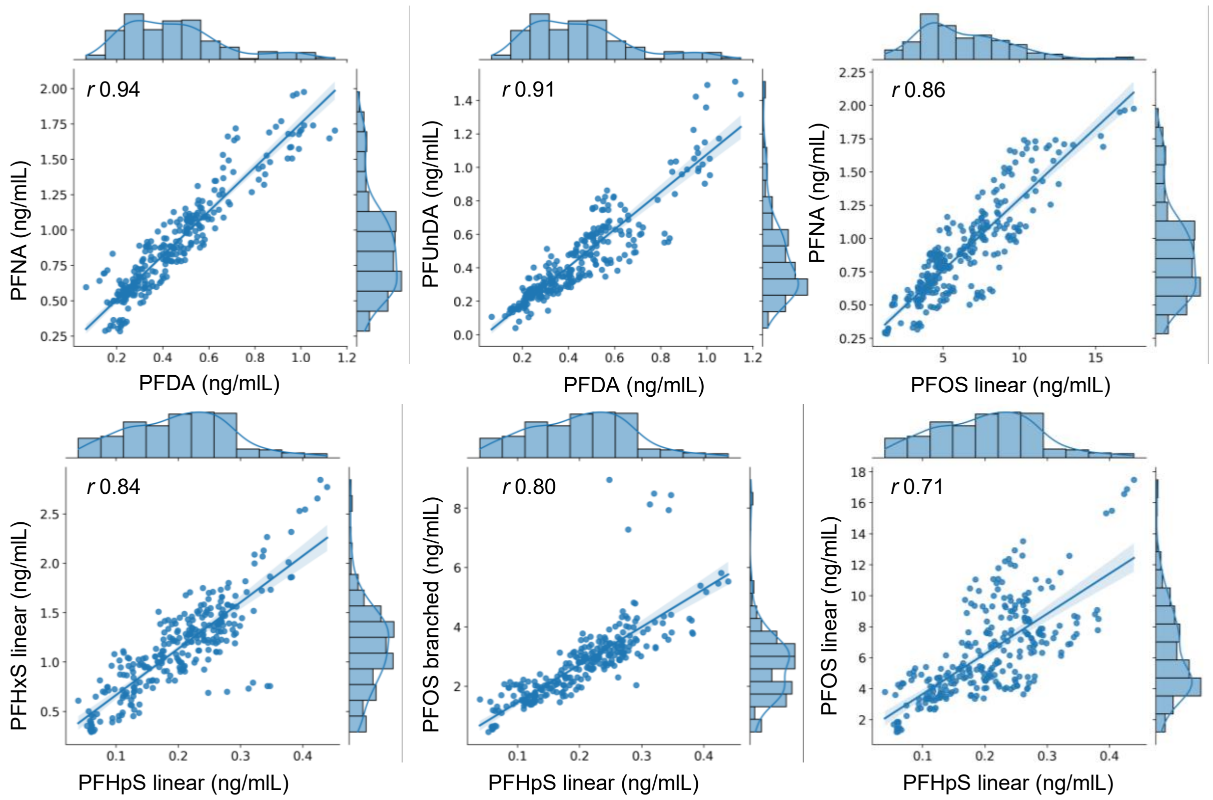

Figure S25: Correlations observed for PFAS targeted analytes. For linear regression the concentrations at all 6 visits of each individual (n= 276) were used and correlation was significant in all cases (p-value < 0.001). The Pearson correlation coefficient (*r*) is shown for each regression along with 95% confidence intervals and analyte distributions in histograms.

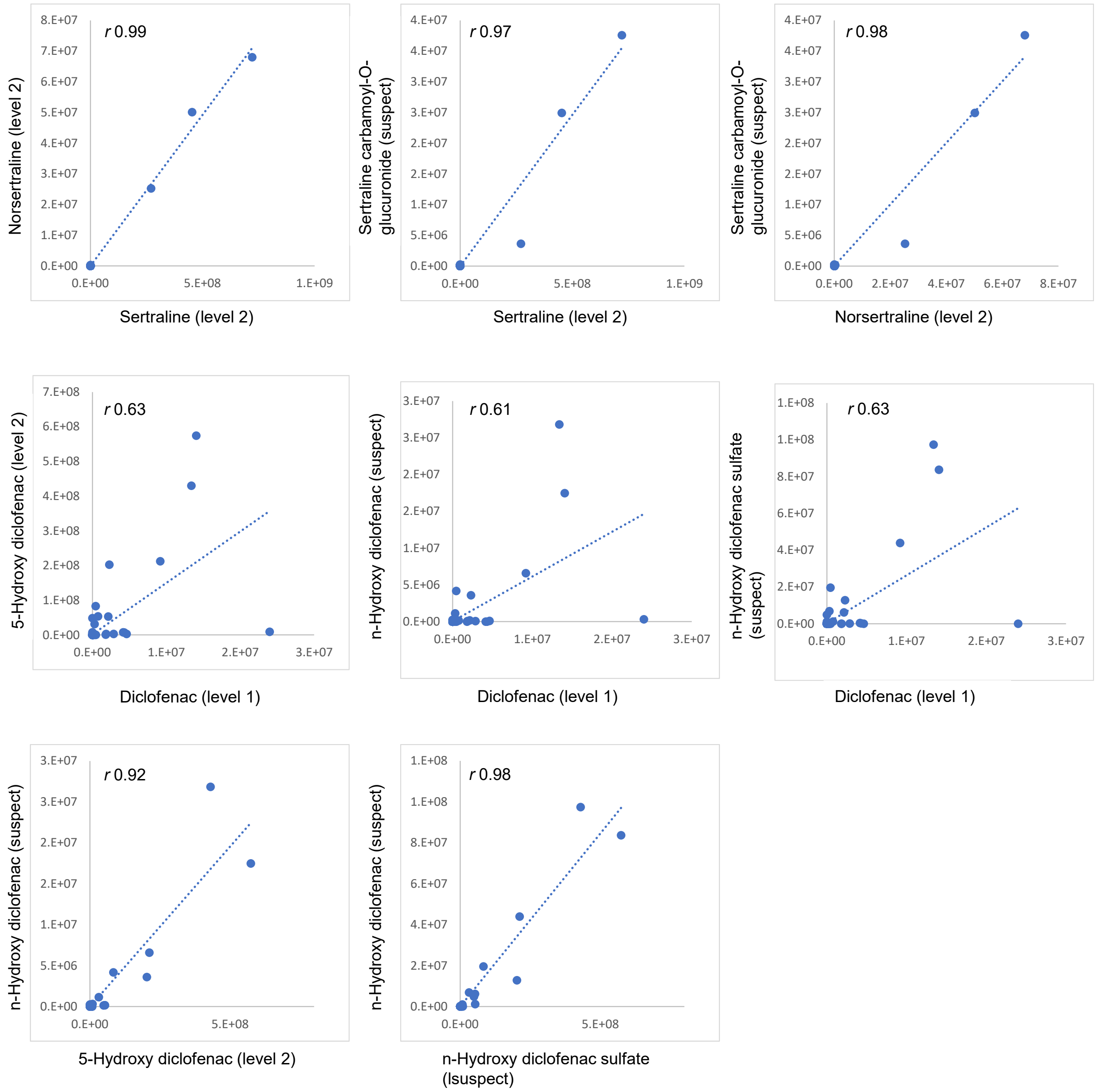

Figure S26: Correlations observed for Level 1 and Level 2 drugs and drug metabolites with suspect drug metabolites (not associated with a spectral library match). For linear regression the normalized areas at all 6 visits of each individual (n= 276) were used and correlation was significant in all cases (p-value < 0.001). The Pearson correlation coefficient (*r*) is shown for each regression.

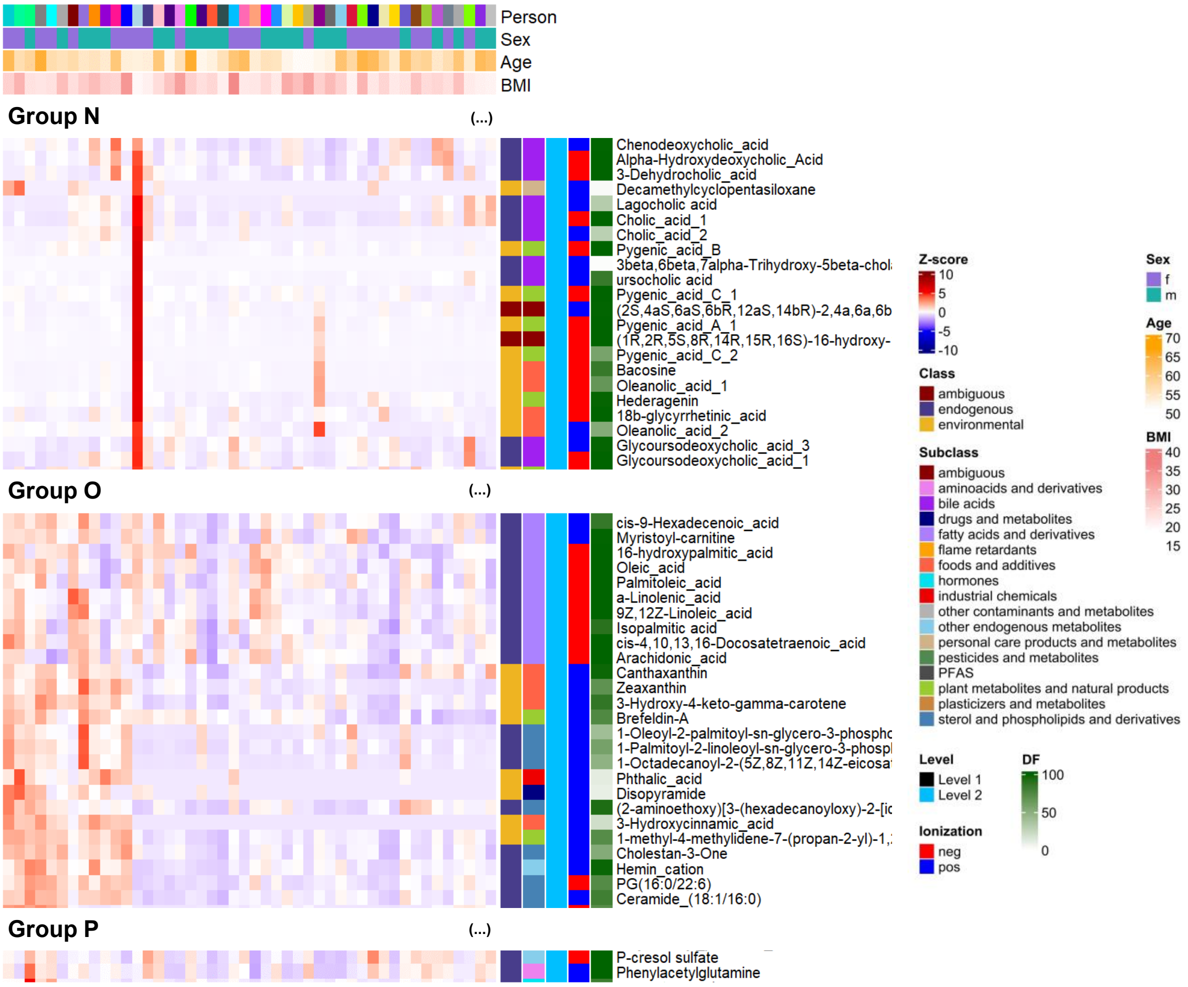

Figure S27: Zoomed-in sections of Hierarchical cluster analysis heatmap, showing the exposome profiles of 46 individuals, each averaged across the 6 clinical visits (full version in Figure 3). The zoomed-in sections show groups involving endogenous metabolites.

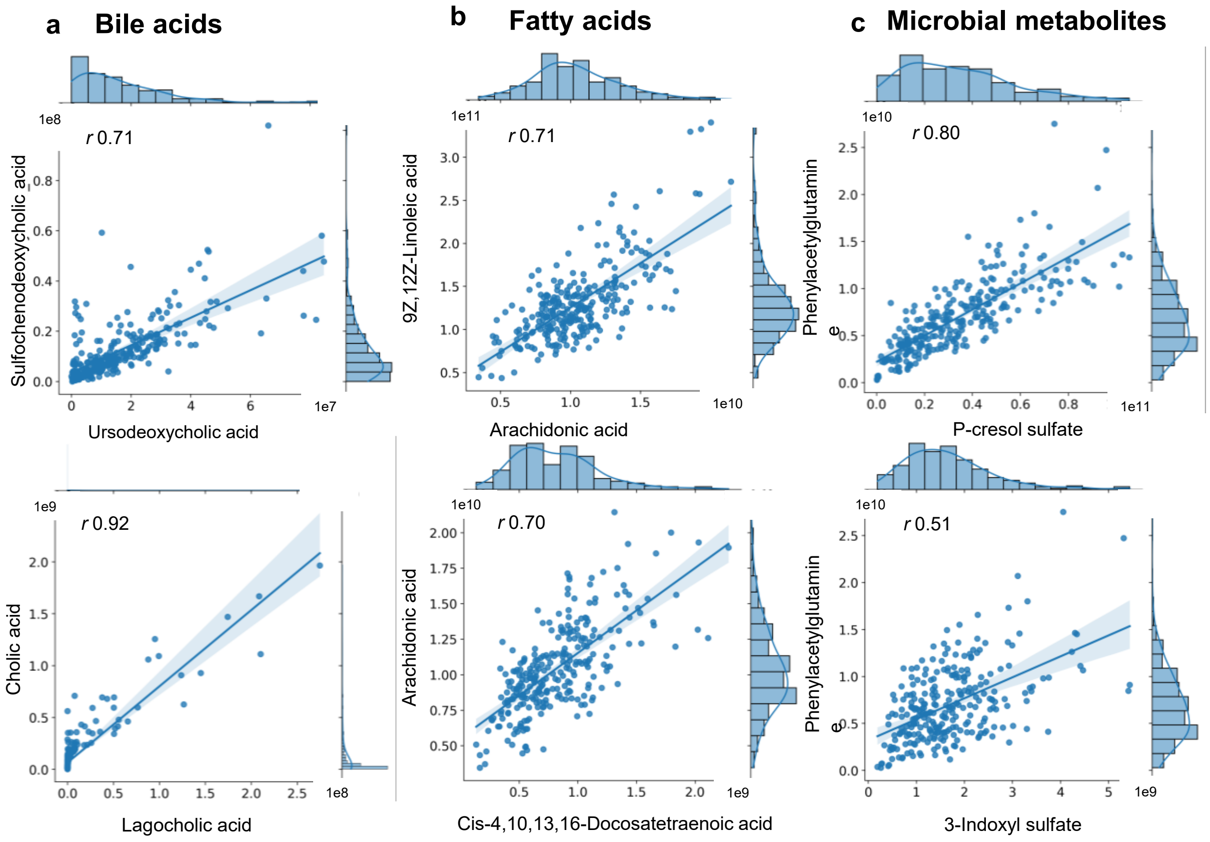

Figure S28: Correlations observed for Level 2 analytes classified as endogenous metabolites. Associations are shown for (a) bile acids (b) fatty acids and (c) microbial metabolites. For linear regression the normalized areas at all 6 visits of each individual (n= 276) were used and correlation was significant in all cases (p-value < 0.001). The Pearson correlation coefficient (*r*) is shown for each regression along with 95% confidence intervals and analyte distributions in histograms.

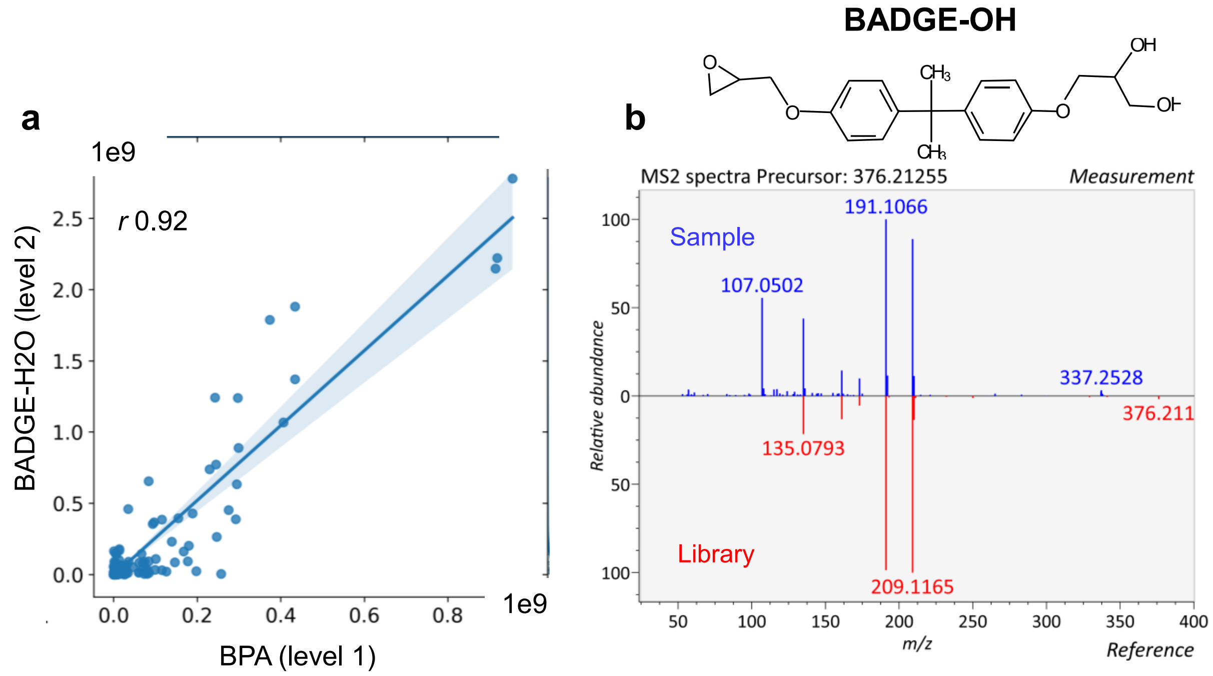

Figure S29: Correlation observed for BADGE-H2O annotated at Level 2 and the targeted analyte BPA. Panel (a) shows the linear regression with 95% confidence interval between BPA and BADGE-H2O, using the normalized areas at all 6 visits of each individual (n= 276). The correlation was significant (p-value < 0.001) and the Pearson correlation coefficient (*r*) is shown on panel (a). Panel (b) shows the structure for BADGE-OH and the corresponding spectral library match.

Table S5. Untargeted molecular discoveries in plasma samples confirmed at confidence Level 1. The table shows chemical name, structure, sources, detection frequency (DF), concentration range among detections (semi-quantification), and previous reports in environmental (Env) or human (Hmn) samples searched in CAS SciFinder[n9] and Blood Exposome Database[10].

Compound	Structure	Source	DF (%)	Detected concentration range (ng/mL)	Previous reports (Env / Hmn)
1,3-Diphenylguanidine	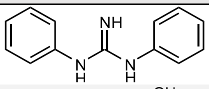	Tire-derived contaminant	83%	0.2-522	Env[11,12], Hmn[13–15]
4-tert-Butylpyrocatechol	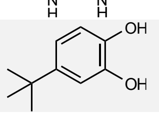	Polymerization inhibitor	92%	N.Q.	Env[16], Hmn: missing
4-Methyl-1H-benzotriazole and 5-Methyl-1H-benzotriazole (coelution) *	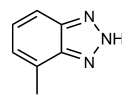	Corrosion inhibitor, ultraviolet stabilizer	9%	3.9-230	Env[17,18], Hmn[19,20]
2,6-Di-tert-butyl-4-nitrophenol	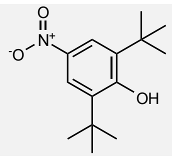	Synthetic antioxidant reaction product	20%	N.Q.	Env[21–23], Hmn: missing
1-Naphthalenesulfonate and 2-Naphthalenesulfonate (close elution) *	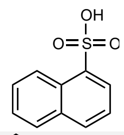	Intermediate in textiles, pharmaceuticals, agrochemicals	13%	0.007-0.050	Env[18,24], Hmn[1]
Triphenyl phosphate	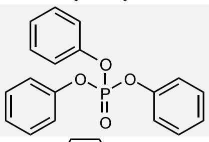	Plasticizer, flame retardant	0.4%	0.1-5.8	Env[25–27], Hmn[28,29]
Triphenylphosphine oxide	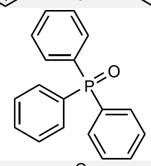	Intermediate in pharmaceuticals	3%	0.04-2.3	Env[30,31], Hmn[13,32]
Diethyl phthalate	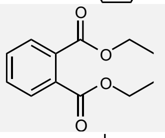	Plasticizer	99.6%	0.05-117	Env[33,34], Hmn[35,36]
Di-2-ethylhexyl phthalate (DEHP)	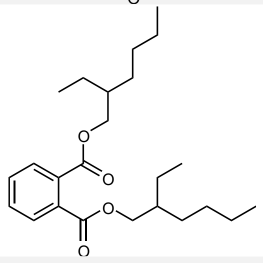	Plasticizer	15%	0.2-34.1	Env[33], Hmn[35,36]
Sodium lauryl sulfate	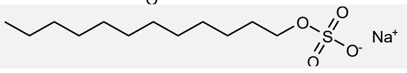	Surfactant	45%	0.3-200	Env[37], Hmn: missing
4-Chlorophenol	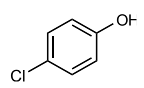	Antiseptic, biodegradation product of chlorobenzene	1%	N.Q.	Env[38,39], Hmn[40]
Chlorothalonil-4-hydroxy	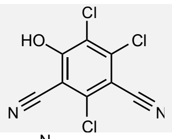	Fungicide transformation product	100%	1.1-11.5	Env[41], Hmn[42–44]
Propiconazole	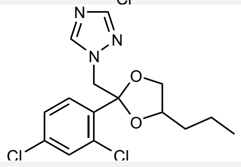	Fungicide	12%	N.Q.	Env[45–47], Hmn: missing
Azelaic acid	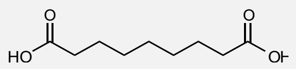	Food component, personal care product	100%	2.5-51.0	Env[48], Hmn[49]
Cyclohexylamine	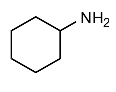	Industrial, food additive metabolite	37%	N.Q.	Env[50], Hmn[51]
N-cinnamoylglycine	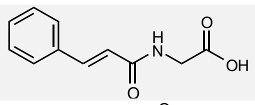	Food component	100%	N.Q.	Env: missing, Hmn[52,53]
3′-Hydroxycotinine	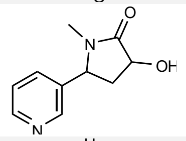	Nicotine metabolite	88%	N.Q.	Env[54], Hmn[55,56]
Indolepropionic acid	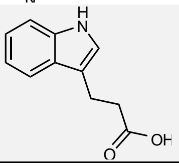	Human microbial metabolite, Plant auxin	100%	N.Q.	Env[48], Hmn[57]

*Only structure of first reported isomer is shown. N.Q. = not quantified.

References

(1) Sdougkou, K.; Xie, H.; Papazian, S.; Bonnefille, B.; Bergdahl, I. A.; Martin, J. W. Phospholipid Removal for Enhanced Chemical Exposomics in Human Plasma. *Environ. Sci. Technol.* **2023**. https://doi.org/10.1021/acs.est.3c00663.

(2) Preindl, K.; Braun, D.; Aichinger, G.; Sieri, S.; Fang, M.; Marko, D.; Warth, B. A Generic Liquid Chromatography−Tandem Mass Spectrometry Exposome Method for the Determination of Xenoestrogens in Biological Matrices. *Anal. Chem.* **2019**, *91* (17), 11334–11342. https://doi.org/10.1021/acs.analchem.9b02446.

(3) Go, Y.-M.; Walker, D. I.; Liang, Y.; Uppal, K.; Soltow, Q. A.; Tran, V.; Strobel, F.; Quyyumi, A. A.; Ziegler, T. R.; Pennell, K. D.; Miller, G. W.; Jones, D. P. Reference Standardization for Mass Spectrometry and High-Resolution Metabolomics Applications to Exposome Research. *Toxicol. Sci.* **2015**, *148* (2), 531–543. https://doi.org/10.1093/toxsci/kfv198.

(4) Inoue, K.; Yamaguchi, A.; Wada, M.; Yoshimura, Y.; Makino, T.; Nakazaw, H. Quantitative Detection of Bisphenol A and Bisphenol A Diglycidyl Ether Metabolites in Human Plasma by Liquid Chromatography-Electrospray Mass Spectrometry. *J Chromatogr B Biomed Sci Appl* **2001**, *765* (2), 121–126. https://doi.org/10.1016/s0378-4347(01)00393-0.

(5) Sajiki, J.; Takahashi, K.; Yonekubo, J. Sensitive Method for the Determination of Bisphenol-A in Serum Using Two Systems of High-Performance Liquid Chromatography. *Journal of Chromatography B: Biomedical Sciences and Applications* **1999**, *736* (1), 255–261. https://doi.org/10.1016/S0378-4347(99)00471-5.

(6) Wang, L.; Liao, C.; Liu, F.; Wu, Q.; Guo, Y.; Moon, H.-B.; Nakata, H.; Kannan, K. Occurrence and Human Exposure of P-Hydroxybenzoic Acid Esters (Parabens), Bisphenol A Diglycidyl Ether (BADGE), and Their Hydrolysis Products in Indoor Dust from the United States and Three East Asian Countries. *Environ. Sci. Technol.* **2012**, *46* (21), 11584–11593. https://doi.org/10.1021/es303516u.

(7) Chang, Y.; Nguyen, C.; Paranjpe, V. R.; Gilliland, F.; Zhang, J. (Jim). Analysis of Bisphenol A Diglycidyl Ether (BADGE) and Its Hydrolytic Metabolites in Biological Specimens by High-Performance Liquid Chromatography and Tandem Mass Spectrometry. *Journal of Chromatography B* **2014**, *965*, 33–38. https://doi.org/10.1016/j.jchromb.2014.06.005.

(8) Tebani, A.; Gummesson, A.; Zhong, W.; Koistinen, I. S.; Lakshmikanth, T.; Olsson, L. M.; Boulund, F.; Neiman, M.; Stenlund, H.; Hellström, C.; Karlsson, M. J.; Arif, M.; Dodig-Crnković, T.; Mardinoglu, A.; Lee, S.; Zhang, C.; Chen, Y.; Olin, A.; Mikes, J.; Danielsson, H.; von Feilitzen, K.; Jansson, P.-A.; Angerås, O.; Huss, M.; Kjellqvist, S.; Odeberg, J.; Edfors, F.; Tremaroli, V.; Forsström, B.; Schwenk, J. M.; Nilsson, P.; Moritz, T.; Bäckhed, F.; Engstrand, L.; Brodin, P.; Bergström, G.; Uhlen, M.; Fagerberg, L. Integration of Molecular Profiles in a Longitudinal Wellness Profiling Cohort. *Nat Commun* **2020**, *11* (1), 4487. https://doi.org/10.1038/s41467-020-18148-7.

(9) *CAS Solutions Login | CAS*. https://www.cas.org/cas-solutions-login (accessed 2023-09-29).

(10) Barupal, D. K.; Fiehn, O. Generating the Blood Exposome Database Using a Comprehensive Text Mining and Database Fusion Approach. *Environ Health Perspect* **2019**, *127* (9), 097008. https://doi.org/10.1289/EHP4713.

(11) Johannessen, C.; Saini, A.; Zhang, X.; Harner, T. Air Monitoring of Tire-Derived Chemicals in Global Megacities Using Passive Samplers. *Environmental Pollution* **2022**, *314*, 120206. https://doi.org/10.1016/j.envpol.2022.120206.

(12) Li, Z.-M.; Kannan, K. Occurrence of 1,3-Diphenylguanidine, 1,3-Di-o-Tolylguanidine, and 1,2,3-Triphenylguanidine in Indoor Dust from 11 Countries: Implications for Human Exposure. *Environ. Sci. Technol.* **2023**, *57* (15), 6129–6138. https://doi.org/10.1021/acs.est.3c00836.

(13) Gil-Solsona, R.; Nika, M.-C.; Bustamante, M.; Villanueva, C. M.; Foraster, M.; Cosin-Tomás, M.; Alygizakis, N.; Gómez-Roig, M. D.; Llurba-Olive, E.; Sunyer, J.; Thomaidis, N. S.; Dadvand, P.; Gago-Ferrero, P. The Potential of Sewage Sludge to Predict and Evaluate the Human Chemical

Exposome. *Environ. Sci. Technol. Lett.* **2021**, *8* (12), 1077–1084. https://doi.org/10.1021/acs.estlett.1c00848.

(14) Li, J.; Zeng, X.; Liang, X.; Tang, S.; Covaci, A.; Ma, X.; Yang, Y.; Chen, D. Gestational Exposure to Plastic Additives and Associations with Placental Function-Related Genes. *Environ. Sci. Technol. Lett.* **2023**, *10* (1), 86–92. https://doi.org/10.1021/acs.estlett.2c00870.

(15) Tang, S.; Sun, X.; Qiao, X.; Cui, W.; Yu, F.; Zeng, X.; Covaci, A.; Chen, D. Prenatal Exposure to Emerging Plasticizers and Synthetic Antioxidants and Their Potency to Cross Human Placenta. *Environ. Sci. Technol.* **2022**, *56* (12), 8507–8517. https://doi.org/10.1021/acs.est.2c01141.

(16) Sheridan, E. A.; Fonvielle, J. A.; Cottingham, S.; Zhang, Y.; Dittmar, T.; Aldridge, D. C.; Tanentzap, A. J. Plastic Pollution Fosters More Microbial Growth in Lakes than Natural Organic Matter. *Nat Commun* **2022**, *13* (1), 4175. https://doi.org/10.1038/s41467-022-31691-9.

(17) Zhu, Q.; Liao, C.; Jiang, G. Occurrence of Human Exposure to Benzothiazoles and Benzotriazoles in Indoor Dust in Suizhou and Beijing, China. *Chem. Res. Chin. Univ.* **2023**, *39* (3), 508–515. https://doi.org/10.1007/s40242-023-3062-9.

(18) Zhao, J.-H.; Hu, L.-X.; Xiao, S.; Zhao, J.-L.; Liu, Y.-S.; Yang, B.; Zhang, Q.-Q.; Ying, G.-G. Screening and Prioritization of Organic Chemicals in a Large River Basin by Suspect and Non-Target Analysis. *Environmental Pollution* **2023**, *333*, 122098. https://doi.org/10.1016/j.envpol.2023.122098.

(19) Li, J.; Zhao, H.; Zhou, Y.; Xu, S.; Cai, Z. Determination of Benzotriazoles and Benzothiazoles in Human Urine by UHPLC-TQMS. *Journal of Chromatography B* **2017**, *1070*, 70–75. https://doi.org/10.1016/j.jchromb.2017.10.045.

(20) Li, X.; Wang, L.; Asimakopoulos, A. G.; Sun, H.; Zhao, Z.; Zhang, J.; Zhang, L.; Wang, Q. Benzotriazoles and Benzothiazoles in Paired Maternal Urine and Amniotic Fluid Samples from Tianjin, China. *Chemosphere* **2018**, *199*, 524–530. https://doi.org/10.1016/j.chemosphere.2018.02.076.

(21) Grigoriadou, A.; Schwarzbauer, J. Non-Target Screening of Organic Contaminants in Sediments from the Industrial Coastal Area of Kavala City (NE Greece). *Water Air Soil Pollut* **2011**, *214* (1), 623–643. https://doi.org/10.1007/s11270-010-0451-8.

(22) Lestido-Cardama, A.; Rodríguez Bernaldo de Quirós, A.; Bustos, J.; Lomo, M. L.; Paseiro Losada, P.; Sendón, R. Estimation of Dietary Exposure to Contaminants Transferred from the Packaging in Fatty Dry Foods Based on Cereals. *Foods* **2020**, *9* (8), 1038. https://doi.org/10.3390/foods9081038.

(23) Still, K. R.; Jung, A. E.; Ritchie, G. D.; Jederberg, W. W.; Wilfong, E. R.; Briggs, G. B.; Arfsten, D. P. Disposition of 2,6-Di-Tert-Butyl-4-Nitrophenol (DBNP), a Submarine Atmosphere Contaminant, in Male Sprague–Dawley Rats. *Environmental Research* **2005**, *98* (3), 363–367. https://doi.org/10.1016/j.envres.2004.08.009.

(24) Bonnefille, B.; Karlsson, O.; Rian, M. B.; Raqib, R.; Parvez, F.; Papazian, S.; Islam, M. S.; Martin, J. W. Nontarget Analysis of Polluted Surface Waters in Bangladesh Using Open Science Workflows. *Environ. Sci. Technol.* **2023**, *57* (17), 6808–6824. https://doi.org/10.1021/acs.est.2c08200.

(25) Onoja, S.; Abdallah, M. A.-E.; Harrad, S. Concentrations, Spatial and Seasonal Variations of Organophosphate Esters in UK Freshwater Sediment. *Emerging Contaminants* **2023**, *9* (3), 100243. https://doi.org/10.1016/j.emcon.2023.100243.

(26) Pomata, D.; Di Filippo, P.; Riccardi, C.; Buiarelli, F.; Marini, F.; Romani, L.; Lucarelli, F.; Pazzi, G.; Galarini, R.; Simonetti, G. Concentrations and Co-Occurrence of 101 Emerging and Legacy Organic Pollutants in the Ultrafine, Fine and Coarse Fractions of Airborne Particulates Associated with Treatment of Waste from Electrical and Electronic Equipment. *Chemosphere* **2023**, *338*, 139443. https://doi.org/10.1016/j.chemosphere.2023.139443.

(27) Tao, F.; Sjöström, Y.; de Wit, C. A.; Hagström, K.; Hagberg, J. Organohalogenated Flame Retardants and Organophosphate Esters from Home and Preschool Dust in Sweden: Pollution Characteristics, Indoor Sources and Intake Assessment. *Science of The Total Environment* **2023**, *896*, 165198. https://doi.org/10.1016/j.scitotenv.2023.165198.

(28) Choo, G.; Ekpe, O. D.; Kim, D.-H.; Oh, J.-E. Human Exposure to Short-Chain Chlorinated Paraffins and Organophosphate Flame Retardants in Relation to Paired Multiple Sources. *Science of The Total Environment* **2023**, *875*, 162681. https://doi.org/10.1016/j.scitotenv.2023.162681.

(29) Liao, K.; Zhao, Y.; Qu, J.; Yu, W.; Hu, S.; Fang, S.; Zhao, M.; Jin, H. Organophosphate Esters Concentrations in Human Serum and Their Associations with Sjögren Syndrome. *Environmental Pollution* **2023**, *331*, 121941. https://doi.org/10.1016/j.envpol.2023.121941.

(30) Faiz, Y.; Zhao, W.; Feng, J.; Sun, C.; He, H.; Zhu, J. Occurrence of Triphenylphosphine Oxide and Other Organophosphorus Compounds in Indoor Air and Settled Dust of an Institute Building. *Building and Environment* **2016**, *106*, 196–204. https://doi.org/10.1016/j.buildenv.2016.06.022.

(31) Besis, A.; Avgenikou, A.; Pantelaki, I.; Serafeim, E.; Georgiadou, E.; Voutsa, D.; Samara, C. Hazardous Organic Pollutants in Indoor Dust from Elementary Schools and Kindergartens in Greece: Implications for Children's Health. *Chemosphere* **2023**, *310*, 136750. https://doi.org/10.1016/j.chemosphere.2022.136750.

(32) Liu, Y.; Zhu, T.; Xie, Z.; Deng, C.; Qi, X.; Hu, R.; Wang, J.; Chen, J. Human Exposure to Chlorinated Organophosphate Ester Flame Retardants and Plasticizers in an Industrial Area of Shenzhen, China. *International Journal of Environmental Research and Public Health* **2022**, *19* (5), 3126. https://doi.org/10.3390/ijerph19053126.

(33) Anake, W. U.; Nnamani, E. A. Levels and Health Risk Assessments of Phthalate Acid Esters in Indoor Dust of Some Microenvironments within Ikeja and Ota, Nigeria. *Sci Rep* **2023**, *13* (1), 11209. https://doi.org/10.1038/s41598-023-38062-4.

(34) Conde-Díaz, A.; Santana-Mayor, Á.; Herrera-Herrera, A. V.; Socas-Rodríguez, B.; Rodríguez-Delgado, M. Á. Assessment of Endocrine Disruptor Pollutants and Their Metabolites in Environmental Water Samples Using a Sustainable Natural Deep Eutectic Solvent-Based Analytical Methodology. *Chemosphere* **2023**, *338*, 139480. https://doi.org/10.1016/j.chemosphere.2023.139480.

(35) Högberg, J.; Hanberg, A.; Berglund, M.; Skerfving, S.; Remberger, M.; Calafat, A. M.; Filipsson, A. F.; Jansson, B.; Johansson, N.; Appelgren, M.; Håkansson, H. Phthalate Diesters and Their Metabolites in Human Breast Milk, Blood or Serum, and Urine as Biomarkers of Exposure in Vulnerable Populations. *Environ Health Perspect* **2008**, *116* (3), 334–339. https://doi.org/10.1289/ehp.10788.

(36) Zhu, J.; Phillips, S. P.; Feng, Y.-L.; Yang, X. Phthalate Esters in Human Milk: Concentration Variations over a 6-Month Postpartum Time. *Environ. Sci. Technol.* **2006**, *40* (17), 5276–5281. https://doi.org/10.1021/es060356w.

(37) Asio, J. R. G.; Garcia, J. S.; Antonatos, C.; Sevilla-Nastor, J. B.; Trinidad, L. C. Sodium Lauryl Sulfate and Its Potential Impacts on Organisms and the Environment: A Thematic Analysis. *Emerging Contaminants* **2023**, *9* (1), 100205. https://doi.org/10.1016/j.emcon.2023.100205.

(38) Tran-Lam, T.-T.; Quan, T. C.; Pham, P. T.; Phung, A.-T. T.; Bui, M. Q.; Dao, Y. H. Occurrence, Distribution, and Risk Assessment of Halogenated Organic Pollutants (HOPs) in Marine Fish Muscle: The Case Study of Vietnam. *Marine Pollution Bulletin* **2023**, *192*, 114986. https://doi.org/10.1016/j.marpolbul.2023.114986.

(39) Persson, Y.; Shchukarev, A.; Öberg, L.; Tysklind, M. Dioxins, Chlorophenols and Other Chlorinated Organic Pollutants in Colloidal and Water Fractions of Groundwater from a Contaminated Sawmill Site. *Environ Sci Pollut Res* **2008**, *15* (6), 463–471. https://doi.org/10.1007/s11356-008-0014-3.

(40) Schmied-Tobies, M. I. H.; Murawski, A.; Schmidt, L.; Rucic, E.; Schwedler, G.; Apel, P.; Göen, T.; Kolossa-Gehring, M. Pentachlorophenol and Nine Other Chlorophenols in Urine of Children and Adolescents in Germany – Human Biomonitoring Results of the German Environmental Survey 2014–2017 (GerES V). *Environmental Research* **2021**, *196*, 110958. https://doi.org/10.1016/j.envres.2021.110958.

(41) Kiefer, K.; Müller, A.; Singer, H.; Hollender, J. New Relevant Pesticide Transformation Products in Groundwater Detected Using Target and Suspect Screening for Agricultural and Urban Micropollutants with LC-HRMS. *Water Research* **2019**, *165*, 114972. https://doi.org/10.1016/j.watres.2019.114972.

(42) Plassmann, M. M.; Fischer, S.; Benskin, J. P. Nontarget Time Trend Screening in Human Blood. *Environ. Sci. Technol. Lett.* **2018**, *5* (6), 335–340. https://doi.org/10.1021/acs.estlett.8b00196.

(43) Pourchet, M.; Narduzzi, L.; Jean, A.; Guiffard, I.; Bichon, E.; Cariou, R.; Guitton, Y.; Hutinet, S.; Vlaanderen, J.; Meijer, J.; Le Bizec, B.; Antignac, J.-P. Non-Targeted Screening Methodology to Characterise Human Internal Chemical Exposure: Application to Halogenated Compounds in Human Milk. *Talanta* **2021**, *225*, 121979. https://doi.org/10.1016/j.talanta.2020.121979.

(44) Krais, A. M.; de Joode, B. van W.; Liljedahl, E. R.; Blomberg, A. J.; Rönnholm, A.; Bengtsson, M.; Cano, J. C.; Hoppin, J. A.; Littorin, M.; Nielsen, C.; Lindh, C. H. Detection of the Fungicide Transformation Product 4-Hydroxychlorothalonil in Serum of Pregnant Women from Sweden and Costa Rica. *J Expo Sci Environ Epidemiol* **2023**, 1–8. https://doi.org/10.1038/s41370-023-00580-8.

(45) Fernández-Fernández, V.; Ramil, M.; Rodríguez, I. Basic Micro-Pollutants in Sludge from Municipal Wastewater Treatment Plants in the Northwest Spain: Occurrence and Risk Assessment of Sludge Disposal. *Chemosphere* **2023**, *335*, 139094. https://doi.org/10.1016/j.chemosphere.2023.139094.

(46) Froger, C.; Jolivet, C.; Budzinski, H.; Pierdet, M.; Caria, G.; Saby, N. P. A.; Arrouays, D.; Bispo, A. Pesticide Residues in French Soils: Occurrence, Risks, and Persistence. *Environ. Sci. Technol.* **2023**, *57* (20), 7818–7827. https://doi.org/10.1021/acs.est.2c09591.

(47) Wolfram, J.; Bub, S.; Petschick, L. L.; Schemmer, A.; Stehle, S.; Schulz, R. Pesticide Occurrence in Protected Surface Waters in Nature Conservation Areas of Germany. *Science of The Total Environment* **2023**, *858*, 160074. https://doi.org/10.1016/j.scitotenv.2022.160074.

(48) Abafe, O. A.; Lawal, M. A.; Chokwe, T. B. Non-Targeted Screening of Emerging Contaminants in South African Surface and Wastewater. *Emerging Contaminants* **2023**, *9* (4), 100246. https://doi.org/10.1016/j.emcon.2023.100246.

(49) Nizioł, J.; Ossoliński, K.; Płaza-Altamer, A.; Kołodziej, A.; Ossolińska, A.; Ossoliński, T.; Nieczaj, A.; Ruman, T. Untargeted Urinary Metabolomics for Bladder Cancer Biomarker Screening with Ultrahigh-Resolution Mass Spectrometry. *Sci Rep* **2023**, *13* (1), 9802. https://doi.org/10.1038/s41598-023-36874-y.

(50) Wang, Y.-Q.; Hu, L.-X.; Zhao, J.-H.; Han, Y.; Liu, Y.-S.; Zhao, J.-L.; Yang, B.; Ying, G.-G. Suspect, Non-Target and Target Screening of Pharmaceuticals and Personal Care Products (PPCPs) in a Drinking Water System. *Science of The Total Environment* **2022**, *808*, 151866. https://doi.org/10.1016/j.scitotenv.2021.151866.

(51) Deng, Y.; Shuai, P.; Wang, H.; Zhang, S.; Li, J.; Du, M.; Huang, P.; Qu, C.; Huang, L. Untargeted Metabolomics for Uncovering Plasma Biological Markers of Wet Age-Related Macular Degeneration. *Aging (Albany NY)* **2021**, *13* (10), 13968–14000. https://doi.org/10.18632/aging.203006.

(52) Santoro, K. L.; Yakah, W.; Singh, P.; Ramiro-Cortijo, D.; Medina-Morales, E.; Freedman, S. D.; Martin, C. R. Acetaminophen and Xenobiotic Metabolites in Human Milk and the Development of Bronchopulmonary Dysplasia and Retinopathy of Prematurity in a Cohort of Extremely Preterm Infants. *The Journal of Pediatrics* **2022**, *244*, 224-229.e3. https://doi.org/10.1016/j.jpeds.2022.01.030.

(53) He, W. J.; Chen, J.; Razavi, A. C.; Hu, E. A.; Grams, M. E.; Yu, B.; Parikh, C. R.; Boerwinkle, E.; Bazzano, L.; Qi, L.; Kelly, T. N.; Coresh, J.; Rebholz, C. M. Metabolites Associated with Coffee Consumption and Incident Chronic Kidney Disease. *Clin J Am Soc Nephrol* **2021**, *16* (11), 1620–1629. https://doi.org/10.2215/CJN.05520421.

(54) Chen, W.-L.; Yu, S.-Y.; Liu, S.-Y.; Lin, S.-C.; Lee, T.-H. Using HRMS Fingerprinting to Explore Micropollutant Contamination in Soil and Vegetables Caused by Swine Wastewater Irrigation.

Science of The Total Environment **2023**, *862*, 160830. https://doi.org/10.1016/j.scitotenv.2022.160830.

(55) Tkalec, Ž.; Codling, G.; Tratnik, J. S.; Mazej, D.; Klánová, J.; Horvat, M.; Kosjek, T. Suspect and Non-Targeted Screening-Based Human Biomonitoring Identified 74 Biomarkers of Exposure in Urine of Slovenian Children. *Environmental Pollution* **2022**, *313*, 120091. https://doi.org/10.1016/j.envpol.2022.120091.

(56) Yakimavets, V.; Qiu, T.; Panuwet, P.; D'Souza, P. E.; Brennan, P. A.; Dunlop, A. L.; Barry Ryan, P.; Boyd Barr, D. Simultaneous Quantification of Urinary Tobacco and Marijuana Metabolites Using Solid-Supported Liquid-Liquid Extraction Coupled with Liquid Chromatography Tandem Mass Spectrometry. *Journal of Chromatography B* **2022**, *1208*, 123378. https://doi.org/10.1016/j.jchromb.2022.123378.

(57) Deng, K.; Xu, J.; Shen, L.; Zhao, H.; Gou, W.; Xu, F.; Fu, Y.; Jiang, Z.; Shuai, M.; Li, B.; Hu, W.; Zheng, J.-S.; Chen, Y. Comparison of Fecal and Blood Metabolome Reveals Inconsistent Associations of the Gut Microbiota with Cardiometabolic Diseases. *Nat Commun* **2023**, *14* (1), 571. https://doi.org/10.1038/s41467-023-36256-y.
